# Health risky behaviors among rural-to-urban migrant workers in China: prevalence, patterns, and association with distal and proximal factors

**DOI:** 10.3389/fpubh.2025.1459661

**Published:** 2025-02-21

**Authors:** Weikai Wang, Mengting Wang, Hong Pan, Wenqian Jian, Li Chen, Yawen Zheng

**Affiliations:** ^1^Department of Psychiatry, Huzhou Third Municipal Hospital, The Affiliated Hospital of Huzhou University, Huzhou, Zhejiang, China; ^2^School of Mental Health, Wenzhou Medical University, Wenzhou, Zhejiang, China; ^3^Lishui Second People's Hospital, Wenzhou Medical University, Lishui, Zhejiang, China

**Keywords:** health risky behaviors, rural-to-urban migrant workers, nomograms, prediction, migrant health

## Abstract

**Background:**

Health Risky Behaviors (HRBs) pose a significant public health challenge, particularly among migrant workers in China who face unfavorable living and working conditions. This study aimed to investigate the prevalence and characteristics of HRBs in rural-to-urban migrant workers, as well as explore factors associated with HRBs from both distal and proximal perspectives.

**Methods:**

A cross-sectional survey involving 2,065 rural-to-urban migrant workers was conducted. Participants completed a structured questionnaire assessing HRBs, distal factors (school dropout, peer victimization, physical neglect/abuse, emotional neglect/abuse) and proximal factors (work burnout, parent-child conflict, adulthood poverty, divorce intention, core self-evaluation). Logistic regression analysis was utilized to identify predictors of HRBs, leading to the development and validation of a prediction model (nomograms) for HRBs among migrant workers. The model's performance was assessed using metrics such as the area under the curve (AUC), calibration curve, receiver operating characteristic (ROC) curve, and decision curve analysis (DCA).

**Results:**

Significant predictors of HRBs included gender, school dropout, peer victimization, abuse/neglect experiences, work burnout, parent-child conflict, adulthood poverty, divorce intention, and core self-evaluation. The developed nomogram showed promising predictive accuracy with an AUC of 0.77 for the training set and 0.76 for the validation set. The calibration curve demonstrated good alignment with the diagonal, and the DCA illustrated the model's utility across different threshold ranges.

**Conclusion:**

This study highlighted a high prevalence of HRBs among migrant workers in China, and the predictive tool developed can be instrumental in informing targeted interventions and policies to address and manage HRBs effectively among this population.

## 1 Introduction

Health Risky Behaviors (HRBs) refer to behaviors such as smoking, excessive alcohol consumption, physical inactivity, unhealthy dietary practices, and risky sexual behaviors, all of which increase the risk of developing chronic diseases, injuries, and mental health problems (1–4). According to the World Health Organization, HRBs are a leading cause of morbidity and mortality globally, accounting for ~60% of all deaths worldwide (5, 6). In China, the prevalence of HRBs is equally alarming. Studies have shown that 27.7% of individuals engage in smoking, 13.7% in excessive alcohol consumption (7–9). A particularly vulnerable group for HRBs in China is the rural-to-urban migrant workers, who numbered an estimated 295.6 million in 2022—over one-third of China's labor force (10). These migrant workers in China face challenges such as poor living conditions, low socioeconomic status (SES), unstable employment, and insufficient social protection (11–13). Such adversities contribute to heightened psychological and social stress, which can predispose them to adopting HRBs. For instance, 6.3% of migrant workers report suicidal thoughts, and 2.5% have attempted suicide (14). Additionally, 32.5% are smokers compared to 22.3% in the general population (15), and 44.2% have experienced physical or psychological intimate partner violence in the past year (16). The implications of HRBs extend beyond individuals' physical and mental health, influencing broader societal and economic systems by affecting labor market dynamics, employment opportunities, and economic stability (17, 18). Given the significant role of migrant workers in contributing to China's GDP, it is crucial to adopt a comprehensive public health strategy focused on primary prevention to address HRBs. This approach aims not only to improve individual wellbeing but also to enhance overall societal and economic resilience.

Understanding the prevalence, patterns, and determinants of HRBs among rural-to-urban migrant workers in China is essential for developing targeted health interventions to improve their health outcomes. Previous research has identified various factors that influence HRBs among migrant workers, including demographic characteristics, work-related conditions, living environments, health literacy, access to healthcare, cultural and social norms, and policy frameworks (19–21). For instance, younger, less educated male migrants were more likely to engage in smoking and excessive alcohol consumption (22), while Xu et al. emphasized the impact of occupational stress and job insecurity on mental health outcomes (23). Berkman et al. also highlighted the importance of limited health knowledge and lack of access to healthcare services in perpetuating unhealthy behaviors (24). However, current research has predominantly examined individual's HRBs in isolation, neglecting the simultaneous occurrence and interplay of multiple risk factors.

Furthermore, previous research has often focused on proximal factors like socioeconomic status and work conditions as key determinants of HRBs among migrant workers (25, 26). However, it is equally important to consider distal factors, such as childhood family experiences and school experiences, which can also influence adult behavior (27, 28). Therefore, integrating both proximal and distal factors in HRBs assessments is essential for developing comprehensive strategies to identify and support migrant workers at risk of engaging in such behaviors.

Proximal-distal theory provides a holistic framework for analyzing HRBs by a complex interplay of proximal and distal factors. In the context of migrant workers, distal factors such as childhood abuse, neglect, family dysfunction, educational disruptions, and childhood abandonment are closely associated with unhealthy behaviors in adulthood (29, 30). Furthermore, proximal factors like personal challenges, the impact of specific events, perceived controllability, and their cumulative effect on resources; work-related stress, and family dynamics can also influence individuals to engage in unhealthy behaviors (31–33). These proximal and distal factors play a crucial role in the development of HRBs among migrant workers, underscoring the significance of incorporating both types of influences into comprehensive prevention strategies. Therefore, this study applies the proximal-distal theory to explore the combined impact of proximal and distal factors on the HRBs among migrant workers.

**Figure 1:**
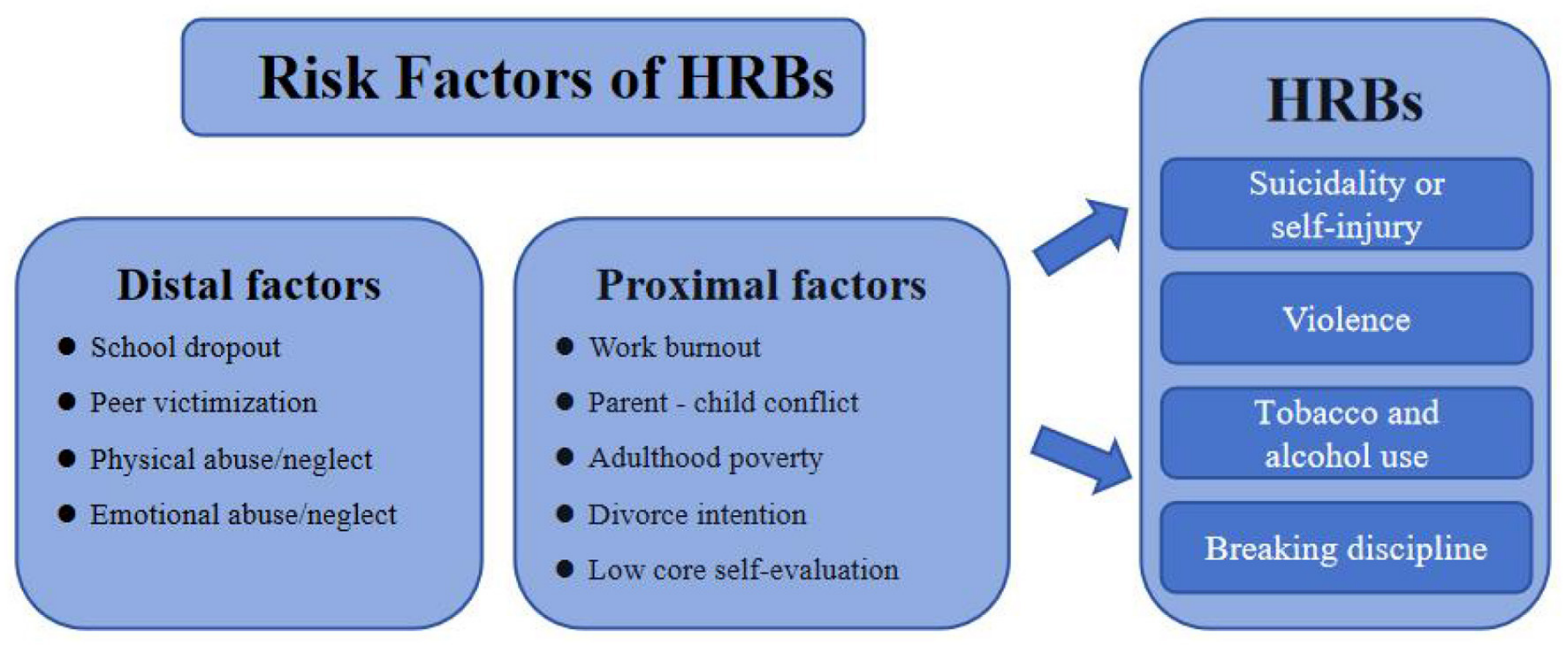
Proximal-distal risk model of HRBs.

The framework for this study, as depicted in Figure 1, highlights how distal and proximal factors together shape the risk of HRBs among rural-to-urban migrant workers. Distal factors, rooted in early life experiences, include school dropout, peer victimization, and physical or emotional abuse/neglect during childhood. These early adversities can have long-lasting effects, shaping an individual's coping mechanisms and increasing vulnerability to HRBs (34, 35). Proximal factors, which exert more immediate influences in adulthood, include work burnout, parent-child conflict, adult poverty, divorce intentions, and low core self-evaluation. These factors directly affect the daily lives of migrant workers, contributing to stress, emotional distress, and maladaptive behaviors (36, 37). For example, work burnout and adult poverty can lead to heightened stress and financial strain, potentially driving individuals to use tobacco or alcohol as coping mechanisms (38, 39). Similarly, parent-child conflict and divorce intentions can intensify emotional turmoil, which may result in violent behaviors or suicidal thoughts (40, 41). Additionally, low core self-evaluation—reflecting an individual's overall self-worth and perceived capabilities—can further exacerbate these issues, making it more challenging for individuals to adopt healthier coping strategies (42). By examining these distal and proximal factors in conjunction, this study aims to provide a comprehensive understanding of the multifaceted influences on HRBs among migrant workers. This integrated approach will facilitate the identification of key areas for intervention and support, ultimately contributing to the development of more effective public health strategies.

Nomograms have become a valuable tool for predicting HRBs, providing a visual representation of risk assessment through the incorporation of demographic characteristics, clinical features, and other relevant variables (43). In the realm of HRBs prevention, nomograms play a crucial role in identifying high-risk individuals, facilitating early intervention and personalized care. While traditionally used in clinical settings, nomograms are now gaining traction in non-clinical research, particularly in predicting HRBs, due to their ability to provide a comprehensive and intuitive approach to evaluating and managing HRBs risk (44).

Therefore, the study aims to (1) examine the prevalence and patterns of HRBs among migrant workers. (2) seeks to identify risk factors at demographic, distal and proximal levels. (3) develop and validate nomograms to predict HRBs among migrant workers based on these factors.

## 2 Method

### 2.1 Participants

For this study, a total of 2,169 rural-to-urban migrant workers were recruited from the Yangtze River Delta region, a plain where the Yangtze River flows into the East China Sea and one of the largest economy-producing areas in China. Participants were required to be 18 years or older, being married with children, and holding an agricultural' hukou status (rural residence) while working as non-agricultural workers in urban areas. Among these, 104 were excluded due to quality issues or logical errors. Ultimately, 2,065 questionnaires were suitable for analysis, resulting in a valid response rate of 95.2%.

The average age of participants was 36.65 years (*SD* = 4.84), with the majority falling between 21 and 43 years old. In terms of gender, females made up 57.1% (*n* = 1,180) while males were 42.9% (*n* = 885) of the sample. Regarding education, 56.6% (*n* = 1,168) had completed secondary education or lower, 37.5% (*n* = 775) had senior secondary education, and a small percentage of 6% (*n* = 122) had a university degree or higher qualifications, and detailed information on characteristics of all participants is shown in Table 1.

**Table 1:** Prevalence and frequency of HRBs among migrant workers (*n* = 2,065).

	**All participants (*n* = 2,065)**	**Health risky behaviors**		***p***	**AOR (95% CI)**	***p***
		**Yes (*n* = 1,664)**	**No (*n* = 401)**			
**Demographic factors**						
Gender				**< 0.001**		**< 0.001**
Male	885	788 (89%)	97 (11%)		1 (ref)	
Female	1,180	876 (74%)	304 (26%)		**0.354 (0.271–0.461)**	
Age				**0.620**		0.703
25 or below	46	36 (78%)	10 (22%)		1 (ref)	
26–35	757	618 (82%)	139 (18%)		1.324 (0.596–2.943)	0.491
36 or above	1,261	1,009 (80%)	252 (20%)		1.214 (0.551–2.674)	0.630
Education level				**0.191**		**< 0.001**
Junior high school or below	1,168	925 (79%)	243 (21%)		1 (ref)	
High school to Secondary	775	638 (82%)	137 (18%)		**1.159 (1.196–2.032)**	**0.001**
College or above	122	101 (83%)	21 (17%)		**2.122 (1.234–3.651)**	**0.007**
**Distal factors (childhood)**						
School dropout				**< 0.001**		**0.016**
No	1,415	1,103 (78%)	312 (22%)		1 (ref)	
Yes	650	561 (86%)	89 (14%)		**1.431 (1.070–1.916)**	
Peer victimization				**0.064**		**0.030**
No	129	112 (87%)	17 (13%)		1 (ref)	
Yes	1,936	1,552 (80%)	384 (20%)		**2.003 (1.068–3.755)**	
Physical abuse/neglect				**< 0.001**		**0.004**
No	1,483	1,133 (76%)	350 (23%)		1 (ref)	
Yes	582	531 (91%)	51 (9%)		**1.783 (1.203–2.643)**	
Emotional abuse/neglect				**< 0.001**		**0.019**
No	1,664	327 (20%)	1,337 (80%)		1 (ref)	
Yes	401	27 (7%)	374 (93%)		**1.792 (1.101–2.918)**	
**Proximal factors (adulthood)**						
Work burnout				**< 0.001**		**0.003**
No	940	672 (71%)	268 (29%)		1 (ref)	
Yes	1,125	992 (88%)	133 (12%)		**1.502 (1.147–1.968)**	
Parent-child conflict				**< 0.001**		**< 0.001**
No	710	482 (68%)	228 (32%)		1 (ref)	
Yes	1,355	1,182 (88%)	173 (12%)		**1.771 (1.355–2.315)**	
Adulthood poverty				**< 0.001**		**0.001**
No	488	327 (67%)	161 (33%)		1 (ref)	
Yes	1,577	1,337 (85%)	240 (15%)		**1.557 (1.193–2.033)**	
Divorce intention				**< 0.001**		**0.003**
No	1,826	1,442 (79%)	21 (21%)		1 (ref)	
Yes	239	222 (93%)	17 (7%)		**2.267 (1.333–3.855)**	
Low core self-evaluation				**< 0.001**		**< 0.001**
No	466	295 (63%)	171 (27%)		1 (ref)	
Yes	1,599	1,369 (86%)	14 (14%)		**1.927 (1.445–2.568)**	

Bold values denote statistically significant improvements (*p* < 0.05).

### 2.2 Procedure

A cross-sectional survey was conducted on rural-to-urban migrant workers using a structured questionnaire distributed online via mobile devices such as iPads or smartphones. Eligible participants were selected following a process consistent with previous studies (16). Initially, a pilot study was conducted with 30 rural-to-urban migrant workers to evaluate the questionnaire's clarity, comprehensiveness, and acceptability. Any duplicate, vague, or inappropriate questions were revised or removed. The study then utilized a multistage probability sampling technique. In the first stage, four cities—Hangzhou, Ningbo, Wenzhou, and Jinhua—were selected from a total of 30 cities in the Yangtze River Delta region, which is situated in the eastern coastal area where the Yangtze River flows into the East China Sea. This region is recognized as one of the most economically vibrant and densely populated areas in China. According to the National Bureau of Statistics, the Yangtze River Delta contributes over 20% of China's GDP and hosts 24% of the nation's rural-to-urban migrant workers (45). These cities were chosen to reflect varying urbanization stages, from developed megacities (Hangzhou, Ningbo) to rapidly growing cities (Wenzhou, Jinhua), ensuring a comprehensive perspective on migration. Additionally, as a pilot region for urban-rural integration, the Yangtze River Delta provides a relevant context for studying migrant workers' experiences. The second stage involved employing a random sampling method in each city. Three districts with high concentrations of rural-urban migrants in each city were randomly chosen, representing inner-city, suburban, and urban fringe areas. Finally, in the third stage, two residential sub-districts with a high density of rural-to-urban migrants in each of the three selected districts were randomly chosen. The sample was stratified by employment sector (construction, manufacturing, service industry, domestic work, and others) to ensure diverse representation. Survey recruitment took place between November 2022 and March 2023, with participation being voluntary and questionnaires remaining anonymous. The study was conducted in accordance with the Helsinki Declaration and received approval from the Ethics Committee of Wenzhou Medical University (approval number: 2022 - 08).

### 2.3 Measurements

#### 2.3.1 Demographic factors

The demographic factors include gender, age, educational level. Educational level is categorized as either junior high school or below, high school to secondary and college or above.

#### 2.3.2 Health risky behaviors

Health risky behaviors in this study were assessed using the 38-item Health Risky Behavior Inventory (3), which covers five main domains: violence, suicidal or self-harm behaviors, tobacco or alcohol use, breaking discipline, and unprotected sex. For our research, we utilized 22 items from five of these domains, excluding unprotected sexual behaviors. Participants indicated the frequency of their engagement in these risky activities over the past year on a scale from 1 (Never) to 5 (Very often), with higher scores indicating greater involvement in HRBs. The instrument has been validated for its reliable psychometric properties in Chinese populations, with a reported Cronbach's alpha coefficient of 0.92 for the overall inventory (46). In our analysis, we used HRBs performed at least once as the threshold for defining HRBs, classifying HRBs as “yes” or “no.”

#### 2.3.3 Distal factors

The study used five risk indicators from childhood. This includes four factors within the family system, such as physical abuse or neglect, emotional abuse or neglect, as well as two factors within the school system, such as school dropout and peer victimization.

##### 2.3.3.1 School dropout

According to Liu et al. (47) and Gaviria and Raphael (48), information of school dropout of migrant workers during compulsory education was collected using a single question: “Did you ever experience dropping out of school before the age of 16?,” “Respondents were required to select either,” “yes” or “no” as their answer.

##### 2.3.3.2 Peer victimization

A single question from the Chinese version of Revised Adverse Childhood Experience Questionnaire [ACEQ-R; (49)] was used to gather information on peer victimization. “The question asked respondents,” “When you were a child, did you ever experience peer victimization?,” with response options of “yes” or “no.”

##### 2.3.3.3 Physical abuse or neglect

Two question from the Chinese version of Revised Adverse Childhood Experience Questionnaire [ACEQ-R; (49)] was used to gather information on physical abuse and neglect. The question asked respondents, “When you were a kid, did your family beat you a lot?,” “When you were a kid, did you often go hungry and wear dirty clothes?” with response options of “yes” or “no.”

##### 2.3.3.4 Emotional abuse or neglect

Two question from the Chinese version of Revised Adverse Childhood Experience Questionnaire [ACEQ-R; (49)] was used to gather information on emotional abuse and neglect. The question asked respondents, “When you were a child, did your family scold you a lot?,” “When you were a child, did you often feel that your family didn't love you?” with response options of “yes” or “no.”

#### 2.3.4 Proximal factors

##### 2.3.4.1 Work burnout

The 15-items Maslach Burnout Inventory-General Survey (MBI-GS) was used to measured work burnout with (50). Respondents rated items on a scale from 0 (never) to 6 (every day). The MBI demonstrated good internal consistency across different countries with alpha values ranging from 0.85 to 0.89 (51–53). In this study, the risk of work burnout was defined using the 25th percentile as the criteria, categorizing individuals into “yes” or “no” for the risk of work burnout.

##### 2.3.4.2 Parent-child conflict

The 12-item parent-child conflict subscale of the parent-child relationship scale (54) was used to assess parent-child conflict. In this scale, participants are asked to respond on a 5-point Likert scale (from “1” = “strongly disagree” to “5” = “strongly agree”). Higher scores indicate greater levels of parent-child conflict. Previous research demonstrates the parent-child conflict subscale has good internal consistency [Coeffcient Alpha = 0.87; (55)]. In this study, we conducted 25th percentile as the risk definition criteria, categorizing individuals into “yes” or “no” for parent-child conflict.

##### 2.3.4.3 Adulthood poverty

The Chinese revised version (56) of the Economic Stress Scale prepared by Wadsworth and Compas uses a 5-point scale with four items, including four dimensions of “food, clothing, housing and transportation (57).” For example: “My family doesn't have enough money to buy new clothes” 1 means “never” and 5 means “always.” Calculate the average score of the four items, the higher the score indicates the greater the adulthood poverty. Previous studies proved good reliability and validity [Coeffcient Alpha = 0.84; (58)]. In this study, we used the 25th percentile as the criteria for defining the risk of poverty, categorizing individuals as either “yes” or “no” for being at risk.

##### 2.3.4.4 Divorce intention

The Chinese Marital Quality Scale (CMQS) with 5 items was employed to measure participants' intentions toward divorce (59). This scale prompts responses on a 4-point scale, ranging from 1 (never) to 4 (recently), where higher scores reflect a stronger inclination toward divorce. The CMQS is validated with a high internal consistency [Coefficient Alpha = 0.79; (60)]. In this study, we conducted 25th percentile as the risk definition criteria, dichotomizing risk of poverty on “yes” or “no.”

##### 2.3.4.5 Core self-evaluation

Core Self-Evaluation scale is a tool for directly assessing core self-evaluation developed by Zenger et al. translated and adapted the core self-evaluation scale based on relevant theories and research (61), creating a version suitable for the Chinese cultural context. The core self-evaluation scale is a unidimensional assessment comprising 10 items, rated on a five-point scale from 1 to 5 to indicate varying levels of agreement, from complete disagreement to complete agreement. Scores range from 10 to 50 points, with higher scores reflecting higher levels of core self-evaluation. The Cronbach's alpha coefficient for the core self-evaluation scale is 0.83, split-half reliability is 0.84, and test-retest reliability over a 3-week interval is 0.82 (62, 63). In this study, we defined the risk of low self-evaluation using the 25th percentile as the cutoff, categorizing individuals into “yes” or “no” for experiencing low self-evaluation.

### 2.4 Statistical analysis

**Figure 2:**
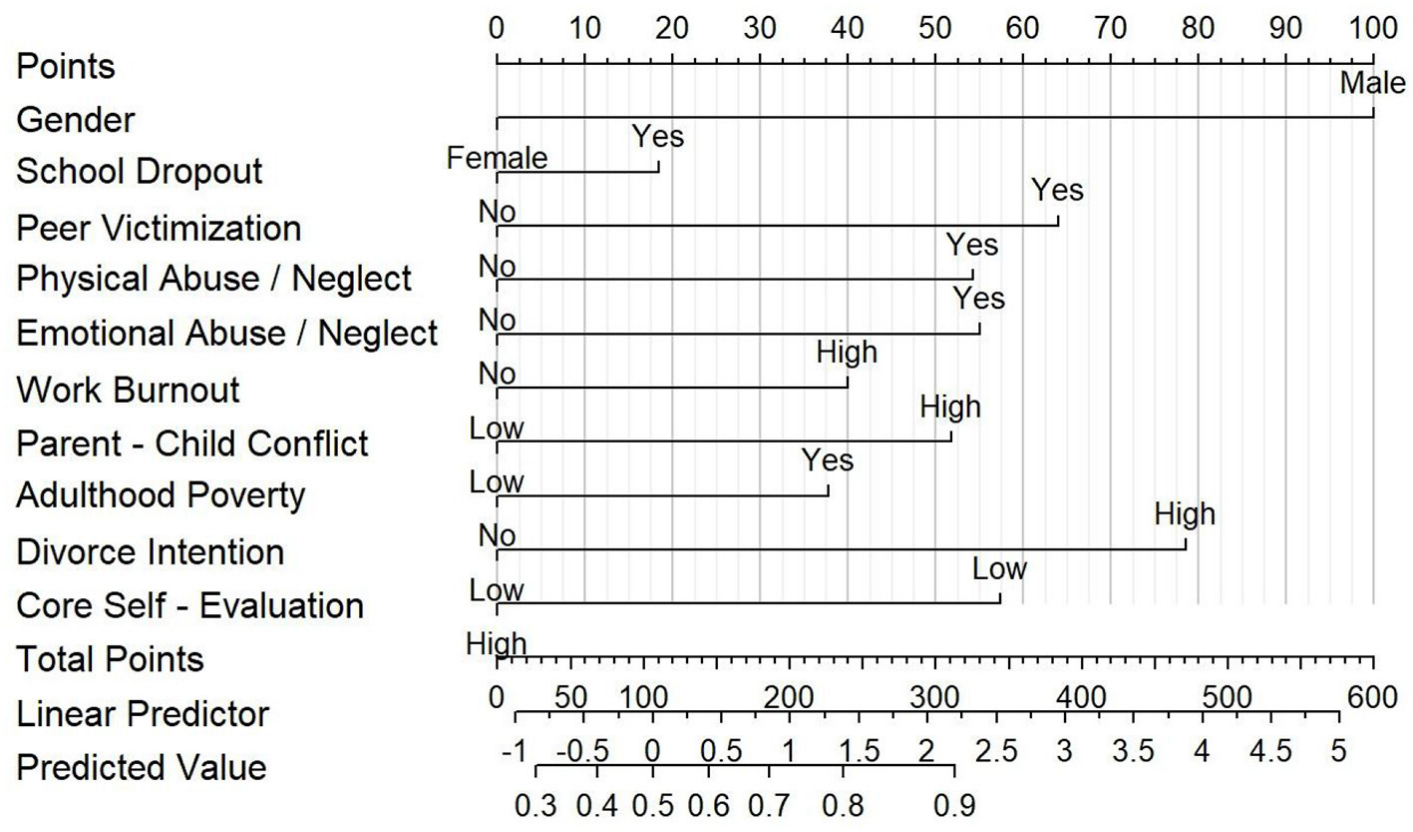
Nomograms of predicting health risky behavior.

Demographic characteristics and key variables of the sample were analyzed using independent samples *t*-tests and ANOVA in SPSS 26. A binary logistic regression model was established with HRBs as the dependent variable and other key variables as independent variables. Model performance was evaluated using significant variables identified through binary logistic regression, which were then incorporated into a nomogram. The nomograms of overall HRBs were established based on statistically significant variables in the binary logistic regression analysis, as depicted in Figure 2. Each variable within this framework carries a designated score, reflecting varying levels of risk. The cumulative risk score, derived by aggregating these individual variable scores, offers a comprehensive assessment. As the aggregate score climbs, so too does the likelihood of an individual exhibiting HRBs, as inferred from the predictive scales situated at the figures' base.

The assessment focused on four key aspects: discrimination, calibration, clinical applicability, and generalization. Discrimination was assessed by calculating the area under the curve (AUC) of the receiver operating characteristic curve, providing a measure of the model's ability to distinguish between different outcomes. For calibration, the model's accuracy in predicting outcomes was evaluated by comparing the predicted values with the observed results. This was visualized through a calibration curve plot, derived from a 1,000 bootstrap resampling procedure. The clinical applicability of the model was assessed using decision curve analysis (DCA), which quantified the net benefits at various threshold probabilities (64). Lastly, the model's generalization was evaluated by examining the nomogram's performance on a separate validation set, ensuring its robustness and applicability in different sample sets. Analyses were conducted in R (version 4.2.3) statistical software. Significance was determined at *p* < 0.05.

## 3 Result

### 3.1 Prevalence and pattern of HRBs among migrant workers

Table 1 presents the prevalence and frequency of four types of HRBs in a sample of married rural-to-urban migrant workers. Out of the 2,065 participants surveyed, a significant majority (80.6%, 95% CI: 78.87%−82.29%) reported involvement in at least one HRB. Specifically, 75.1% (95% CI: 73.19%−76.93%) reported engaging in violent behavior, followed by tobacco and alcohol use (50.2%, 95% CI: 48.01%−52.33%), breaking discipline (21.6%, 95% CI: 19.87%−23.42%), and suicidality or self-injury (16.0%, 95% CI: 14.45%−17.61%). The analysis also reveals patterns of co-occurrence among different HRBs, with tobacco and alcohol use and violence being the most common combination (45.0%, 95% CI: 42.84%−47.13%). Violence occurring alone was reported by 27.31% (95% CI: 25.39%−29.23%), while breaking discipline without other behaviors was rare (0.2%, 95% CI: 0.00%−0.38%) as seen in Table 2. Importantly, within this group, suicidality or self-injury and tobacco/alcohol use were not typically reported in isolation, emphasizing the interconnected nature of risk factors and the multifactorial nature of HRBs among this population.

**Table 2:** Prevalence and co-occurrence of HRBs.

**Types of HRBs**	**Number %**	**95% CI**
At least one of HRBs	1,664 (80.58%)	78.87%−82.29%
SS	331 (16.03%)	14.45%−17.61%
V	1,550 (75.06%)	73.19%−76.93%
TA	1,036 (50.17%)	48.01%−52.33%
BD	447 (21.65%)	19.87%−23.42%
SS+V	320 (15.50%)	13.94%−17.06%
SS+TA	394 (19.08%)	17.39%−20.77%
SS+BD	231 (11.19%)	9.83%−12.55%
V+TA	929 (44.99%)	42.84%−47.13%
V+BD	433 (20.97%)	19.21%−22.72%
BD+TA	410 (19.85%)	18.13%−21.58%
SS+V+TA	287 (13.90%)	12.41%−15.39%
V+BD+TA	399 (19.32%)	17.62%−21.02%
SS+V+BD	228 (11.04%)	9.69%−12.39%
SS+TA+BD	222 (10.75%)	9.41%−12.09%
SS+TA+BD+V	218 (10.56%)	9.23%−11.88%

SS, suicidality or self-injury; V, violence; TA, tobacco and alcohol use; BD, breaking discipline.

### 3.2 Factors associated with HRBs among migrant workers

#### 3.2.1 Factors associated with overall HRBs

The study in Table 1 reveals that female married migrant workers are significantly less likely to engage in HRBs than male workers (AOR 0.35, 95% CI: 0.27–0.46). Furthermore, married migrant workers with a high school to secondary education level have higher odds of participating in HRBs (AOR 1.16, 95% CI: 1.20–2.03), while those with a college education or higher have lower odds (AOR 0.58, 95% CI: 0.39–0.88), when compared to workers with a junior high school education or less.

From the proximal factors, respondents who reported higher work burnout (AOR 1.50, 95% CI: 1.48–1.97) were more likely to report HRBs. Similarly, married migrant workers who faced a high level of parent-child conflict were more likely to experience more HRBs (AOR 1.77, 95% CI: 1.36–2.32). Those with high divorce intentions had almost double the risk of HRBs (AOR 2.27, 95% CI: 1.33–3.85). Conversely, individuals with higher core self-evaluation were less likely to report HRBs compared to those with low core self-evaluation (AOR 1.93, 95% CI: 1.44–2.57). On Additionally, respondents experiencing poverty were significantly more at risk for HRBs (AOR 1.56, 95% CI: 1.19–2.03).

From the distal factors, individuals who dropped out of school during childhood were more likely to engage in HRBs (AOR 1.43, 95% CI: 1.07–1.92). Furthermore, married migrant workers who had experienced peer victimization had a higher likelihood of engaging in HRBs (AOR 2.00, 95% CI: 1.06–3.76). Additionally, when compared to those from supportive families, married migrant workers who experienced physical abuse or neglect (AOR 1.78, 95% CI: 1.20–2.64) or emotional abuse or neglect (AOR 1.79, 95% CI: 1.10–2.91) were almost twice as likely to exhibit HRBs.

#### 3.2.2 Factors associated with suicidality or self-injury

In the regression model of suicidality or self-injury, as shown in Supplementary Table 1, female had a significantly decreased AOR for suicidality or self-injury compared with male (AOR 0.58, 95% CI: 0.44–0.76). From the proximal factors, parent-child conflict (AOR 2.52, 95% CI: 1.63–3.88) and work burnout (AOR 2.20, 95% CI: 1.58–3.10) significantly predicted suicidality or self-injury. Also, migrant workers with high divorce intentions had over 4-fold the risk of suicidality or self-injury (AOR 4.54, 95% CI: 3.29–6.27). Respondents with lower core self-evaluation (AOR 5.61, 95% CI: 2.40–13.12) were more likely to report HRBs, while those experiencing adulthood poverty (AOR 2.17, 95% CI: 1.31–3.60) were more likely to engage in suicidality or self-injury. From the distal factors, married migrant workers who experienced physical abuse or neglect (AOR 2.52, 95% CI: 1.63–3.89) had more than twice the risk of suicidality or self-injury.

#### 3.2.3 Factors associated with violence

In the regression model of violence, as shown in Supplementary Table 2, female married migrant workers were less likely to report violence compared to male migrant workers (AOR 0.69, 95% CI: 0.55–0.86). Those with higher education levels (AOR 1.63, 95% CI: 1.29–2.07; AOR 2.65, 95% CI: 1.60–4.40) were more likely to engage in violence. From the proximal factors, Married migrant workers with high work burnout (AOR 1.41, 95% CI: 1.10–1.80) or low core self-evaluation (AOR 1.89, 95% CI: 1.44–2.47) were more likely to report violence behavior. In the same way, married migrant workers who had a high parent-child conflict problem (AOR 1.78, 95% CI: 1.40–2.27) or high divorce intention (AOR 2.01, 95% CI: 1.29–3.14) were more likely to participant in violence. It is worthwhile to note that adulthood poverty (AOR 1.89, 95% CI: 1.45–2.47) significantly predicted violence. From the distal factors, respondents who suffered school dropout in childhood (AOR 1.47, 95% CI: 1.14–1.90) were more likely to report current participation in violence. Meanwhile, participants who had experienced peer victimization were more likely to be linked to violence (AOR 1.75, 95% CI: 1.01–3.04). Furthermore, married migrant workers who reported instances of physical abuse or neglect (AOR 1.70, 95% CI: 1.22–2.37) and emotional abuse or neglect (AOR 1.74, 95% CI: 1.15–2.62) exhibited a significantly higher likelihood of engaging in violence.

#### 3.2.4 Factors associated with tobacco and alcohol use

In the regression model of violence, as shown in Supplementary Table 3, female married migrant workers were significantly less likely to report tobacco and alcohol use compared to their male counterparts (AOR 0.10, 95% CI: 0.07–0.12). Among the proximal factors, individuals who reported a high level of work burnout were significantly more likely to report tobacco and alcohol use (AOR 1.69, 95% CI: 1.13–2.14). Similarly, married migrant workers who reported a high level of Parent-Child Conflict were also more likely to experience higher levels of tobacco and alcohol use (AOR 1.73, 95% CI: 1.34–2.22). Married migrant workers with high divorce intentions had nearly twice the risk of tobacco and alcohol use (AOR 2.22, 95% CI: 1.58–3.12). Those with high core self-evaluation were less likely to report tobacco and alcohol use (AOR 1.38, 95% CI: 1.03–1.87). In addition, adulthood poverty was identified as a significant risk factor for tobacco and alcohol use (AOR 1.79, 95% CI: 1.37–2.35). From the distal factors, married migrant workers who experienced emotional abuse or neglect had nearly double the risk of tobacco and alcohol use (AOR 1.74, 95% CI: 1.15–2.62).

#### 3.2.5 Factors associated with breaking discipline

In the regression model of violence, as shown in Supplementary Table 4, female married migrant workers were significantly less likely to report breaking discipline compared to their male (AOR 0.69, 95% CI: 0.55–0.86). From the proximal factors, respondents experiencing a high degree of work burnout (AOR 1.54, 95% CI: 1.17–2.03) were more likely to report current experiences of breaking discipline. In the same way, married migrant workers who had a high parent-child conflict problem were more likely to experience higher breaking discipline (AOR 2.16, 95% CI: 1.55–3.01). Also, married migrant workers with high divorce intentions had nearly twice the risk of breaking discipline compared to those with lower divorce intentions (AOR 2.79, 95% CI: 2.03–3.85). Furthermore, compared with respondents with low core self-evaluation, those with high core self-evaluation (AOR 1.93, 95% CI: 1.25–2.97) were less likely to report breaking discipline. On the other hand, respondents who experienced adulthood poverty (AOR 1.74, 95% CI: 1.19–2.54) were identified as significant risk factors for breaking discipline. From the distal factors, married migrant workers who experienced physical abuse or neglect (AOR 1.70, 95% CI: 1.22–2.37) and emotional abuse or neglect (AOR 1.74, 95% CI: 1.15–2.62) had nearly twice the risk of breaking discipline.

### 3.3 Nomograms and validation

#### 3.3.1 Nomograms and validation of overall HRBs

**Figure 3:**
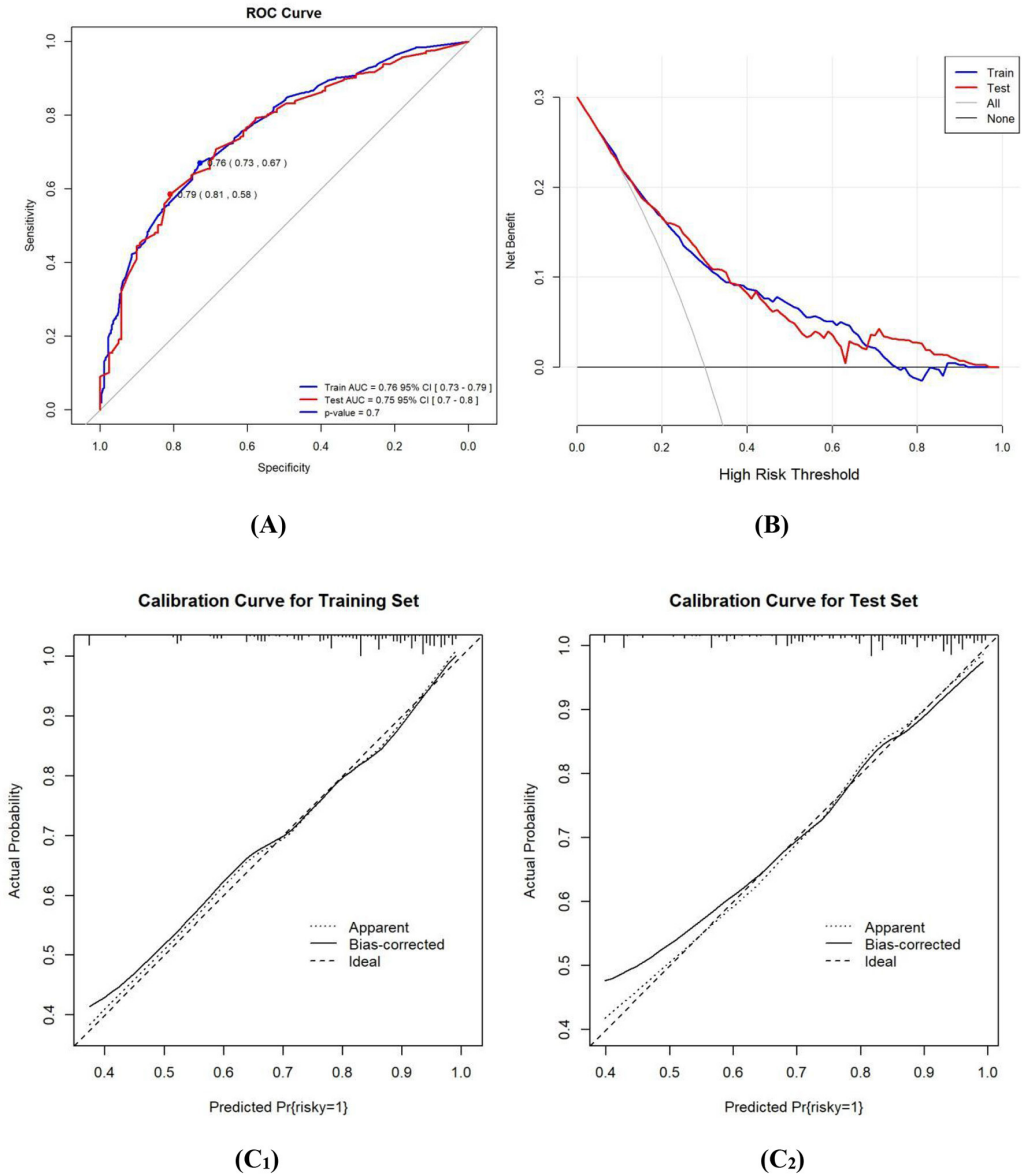
**(A)** ROC curves of the study's generated nomogram for predicting the probability of HRBs. **(B)** DCA for predicting the probability of HRBs nomogram. **(C)** Calibration curves of the nomogram for predicting the probability of HRBs; **(C**_**1**_**)** for the training set; **(C**_**2**_**)** for the internal validation set.

The nomogram's performance of overall HRBs, indicated by the AUC, was found to be 0.78 for the training set and 0.76 for the validation set, with 95% CI of 0.75–0.80 and 0.71–0.81, respectively (as seen in Figure 3). For the training set, the sensitivity was 0.80 and specificity was 0.65. The Youden index stood at 0.45, and the optimal cutoff value was calculated to be 0.83. The calibration curves for both the training and validation sets (training: C = 0.77; validation: C = 0.78), demonstrated a bias correction close to the ideal line, suggesting a strong concordance between the predicted and actual outcomes. Moreover, the Hosmer-Lemeshow test yielded a *p*-value of 0.63, indicating a good fit for the model. The DCA, revealed that the nomogram predictions provided more net benefits across threshold probabilities ranging from 9% to 61%. These findings suggest that the developed nomogram exhibits robust discrimination, calibration, and clinical usefulness for the evaluated risk.

#### 3.3.2 Nomograms and validation of suicidality or self-injury

The nomograms of suicidality or self-injury were established based on statistically significant variables in the binary logistic regression analysis (Supplementary Figure 1). AUC was found to be 0.73 for the training set and 0.74 for the validation set, with 95% Confidence Intervals of 0.71–0.78 and 0.69–0.78, respectively. For the training set, the sensitivity was 0.62 and specificity was 0.76, the Youden index stood at 0.37. Moreover, the Hosmer-Lemeshow test confirmed the model's adequacy (*p* > 0.05). The DCA, as shown in Supplementary Figure 2, revealed that the nomogram predictions provided more net benefits across threshold probabilities ranging from 8% to 82%.

#### 3.3.3 Nomograms and validation of violence

The nomograms of violence were established based on statistically significant variables in the binary logistic regression analysis (Supplementary Figure 3). AUC was found to be 0.76 for the training set and 0.75 for the validation set, with 95% Confidence Intervals of 0.73–0.79 and 0.7–0.8, respectively. For the training set, the sensitivity was 0.59 and specificity was 0.81, the Youden index stood at 0.39. Moreover, the Hosmer-Lemeshow test confirmed the model's adequacy (*p* > 0.05). DCA, as shown in Supplementary Figure 4, revealed that the nomogram predictions provided more net benefits across threshold probabilities ranging from 13% to 59%.

#### 3.3.4 Nomograms and validation of tobacco and alcohol use

The nomograms of tobacco and alcohol use were established based on statistically significant variables in the binary logistic regression analysis (Supplementary Figure 5). AUC was found to be 0.74 for the training set and 0.73 for the validation set, with 95% Confidence Intervals of 0.71–0.77 and 0.68–0.78, respectively. For the training set, the sensitivity was 0.76 and specificity was 0.6, the Youden index stood at 0.36. Moreover, the Hosmer-Lemeshow test confirmed the model's adequacy (*p* > 0.05). The DCA revealed that the nomogram predictions provided more net benefits across threshold probabilities ranging from 9% to 93% (Supplementary Figure 6).

#### 3.3.5 Nomograms and validation of breaking discipline

The nomograms of breaking discipline were established based on statistically significant variables in the binary logistic regression analysis (Supplementary Figure 7). AUC was found to be 0.76 for the training set and 0.76 for the validation set, with 95% Confidence Intervals of 0.73–0.79 and 0.71–0.8, respectively. For the training set, the sensitivity was 0.70 and specificity was 0.73, the Youden index stood at 0.43. Moreover, the Hosmer-Lemeshow test confirmed the model's adequacy (*p* > 0.05). The DCA revealed that the nomograms predictions provided more net benefits across threshold probabilities ranging from 8% to 72% (Supplementary Figure 8).

## 4 Discussion

This study estimated the prevalence, frequency and patterns of four types of HRBs in a large sample of typical Chinese married rural to urban migrant workers, and identified the risk factors for HRBs from both distal and proximal perspectives.

The study found that 80.9% of migrant workers reported experiencing HRBs at least once violence (75.1%) was the most commonly reported type, followed by tobacco and alcohol use (50.7%), breaking discipline (22.2%), and suicidality or self-injury (16.6%). This finding confirms the national study and enhances our comprehension of violence behavior as the most common form of HRBs (65). Additionally, it highlights a significant social concern: the high percentage of aggressive behavior indicates the severe difficulties that the migrant worker community faces. Its consequences are multifaceted, affecting individual mental health, interpersonal relationships and social stability. Therefore, it is important for the government and society to prioritize the protection of the rights and interests of migrant workers, reducing their economic pressures and life difficulties.

Additionally, this study found a high degree of co-morbidity between four HRBs, particularly violence and smoking or alcohol consumption, which is consistent with international studies by Chen et al. (66) and Mischel et al. (67). According to self-regulation theory (68), individuals may resort to unhealthy coping mechanisms, such as smoking, drinking alcohol, or aggression, when confronted with stress and challenges (69). Although these strategies may provide temporary relief from negative emotions, they can result in severe mental and physical health issues in the long run. Therefore, it is important to consider the multiple risk factors that individuals face and their interactions through complex psychosocial mechanisms when developing effective public health strategies.

The third important finding of the study is that some socio-demographic, distal and proximal factors are significantly associated with an increased or decreased likelihood of HRBs among married rural-to-urban migrant workers.

At the demographic level, education level and gender play a significant role in HRBs. Research has consistently shown that males are more likely than females to engage in risky behaviors, including smoking, alcohol consumption, and violence. This trend has been observed across different cultural contexts (70, 71). The higher prevalence of risky behaviors among males could be linked to socio-cultural norms that discourage men from openly expressing vulnerability and emotions. These suppressed emotions may manifest in behaviors such as substance abuse and aggression (72, 73). Additionally, higher educational attainment is correlated with a reduced likelihood of engaging in high-risk behaviors, aligning with findings from previous research (74).

At the proximal level, work burnout, family conflicts, poverty, divorce intention, and low core self-evaluation, were significant predictors of HRBs among migrant workers. Migrant workers often face demanding and stressful working conditions, leading to feelings of burnout and an increased likelihood of engaging in HRBs as a coping mechanism (75). Separation from family members and disrupted family structures can result in strained relationships and emotional distress, which may also contribute to the adoption of HRBs (76). Financial strain and economic hardship, common among migrant workers, can create significant stress and anxiety, further increasing the risk of HRBs (77). Marital problems and the potential dissolution of a marriage can lead to feelings of despair, hopelessness, and depression, which may also increase the likelihood of engaging in HRBs. Lastly, migrant workers with low core self-evaluation may struggle with feelings of inadequacy, low self-esteem, and self-doubt (78), which can contribute to the adoption of HRBs as a means to cope with these negative emotions. These findings emphasize the need for comprehensive interventions that address the specific challenges faced by migrant workers to reduce the risk of HRBs and promote better health outcomes.

At the distal level, school dropout, peer victimization, and experiences of abuse/neglect during childhood were all significant factors in the development of health risk behaviors (HRBs). Dropping out of school can limit access to education and employment opportunities, leading to financial instability and stress (79). Peer victimization can have lasting negative effects on mental health and self-esteem (80–82). Similarly, childhood experiences of abuse and neglect can have severe and long-lasting impacts on mental and emotional wellbeing, contributing to maladaptive coping strategies and difficulties in forming healthy relationships (83, 84). Recognizing the importance of early life experiences and psychosocial resources in assessing and intervening in HRBs is crucial for improving health outcomes among migrant workers. Providing mental health services, educational opportunities, and social support networks to address these underlying factors can help migrant workers overcome challenges stemming from their early life experiences and develop healthier coping mechanisms.

The nomogram developed in this study showed promising predictive accuracy, with an AUC of 0.77 for the training set and 0.76 for the validation set. This indicates that the model has good discriminative ability to distinguish between migrant workers who engage in HRBs and those who do not. The calibration curve demonstrated good alignment with the diagonal, suggesting that the model's predictions were accurate and reliable. The DCA illustrated the model's utility across different threshold ranges, indicating that it can be a useful tool for informing targeted interventions and policies to address HRBs among migrant workers.

### 4.1 Implication

This study has significant implications for research, policy, and practice in addressing HRBs among migrant workers. The findings highlight the importance of conducting comprehensive HRB assessments that consider both proximal and distal factors, including early life experiences and psychosocial resources. To effectively address these risk factors, interventions should be tailored to the specific needs of migrant workers and implemented at multiple levels. Policymakers should prioritize the development of policies that tackle the root causes of HRBs, including enhancing working conditions, ensuring access to affordable healthcare, and providing economic support to migrant workers. Supportive policies, such as affordable housing, health insurance, and educational opportunities, can alleviate the economic and psychological pressures faced by these workers.

Interventions should be customized to address the specific needs of migrant workers through the provision of mental health services, educational opportunities, and social support networks. At the individual level, accessible mental health services, including counseling and therapy, can help manage stress and emotional distress, while cognitive-behavioral therapy (CBT) and health education programs can improve coping mechanisms and reduce unhealthy behaviors. Self-management tools, such as apps, can help individuals monitor and manage their health behaviors. At the family level, counseling services can address conflicts and improve family dynamics, and support programs can provide economic assistance and psychological support to help families cope with stress. Parent-child activities can enhance communication and reduce conflicts. At the societal level, governments can implement supportive policies, such as affordable housing and health insurance, to alleviate economic and psychological pressures. Community programs can provide mental health services and social support, and workplace interventions, including flexible work arrangements and stress management workshops, can help employees cope with work stress. Economic support and early intervention programs can address childhood abuse and neglect, providing resources to vulnerable families to prevent long-term negative outcomes.

By integrating these interventions at the individual, family, and societal levels, public health strategies can effectively address the multiple risk factors and their interactions, promoting healthier coping mechanisms and reducing the prevalence of HRBs among migrant workers. Cross-sectoral collaboration among healthcare, education, social services, and labor sectors is crucial for creating and implementing comprehensive, evidence-based interventions that effectively address HRBs in this vulnerable population.

### 4.2 Strengths and limitations

Several limitations should be considered when interpreting the findings of this study. The cross-sectional design prevents establishing causal relationships between HRBs and the identified predictors. Longitudinal studies are necessary to explore the temporal connections between these variables. Additionally, the self-reported data may have introduced social desirability bias, potentially leading to an underestimation of the true prevalence of HRBs among migrant workers. Moreover, the study sample was restricted to rural-to-urban migrant workers in the Yangtze River Delta region, which could restrict the generalizability of the findings to other regions and populations.

Despite these limitations, our study has several strengths. To our knowledge, this is the first study to investigate the prevalence, patterns, and multifactorial determinants of HRBs among rural-to-urban migrant workers in China using a proximal-distal framework. Additionally, the large sample size and the development and validation of a nomogram for predicting HRBs among migrant workers contribute to the study's novelty and significance.

## 5 Conclusion

This study emphasizes the widespread occurrence of HRBs among migrant workers in China and identifies important predictors from both distant and close viewpoints. The nomogram created in this study can be valuable in guiding specific interventions and policies aimed at addressing and controlling HRBs among this group. Future research should prioritize longitudinal studies to investigate the chronological connections between HRBs and their predictors, along with interventions aimed at reducing HRBs among migrant workers.

## Data Availability

All data used and/or analyzed in the present study are available from the corresponding author on reasonable request. They are not publicly available, in accordance with the Ethics Review Authority.
