# Supplementary material for: Health risky behaviors among rural-to-urban migrant workers in China: prevalence, patterns, and association with distal and proximal factors
Source: Front Public Health. 2025 Feb 21;13:1459661. doi: 10.3389/fpubh.2025.1459661 (PMC11885242; doi:10.3389/fpubh.2025.1459661)
Supplement: Supplementary file 1 [file Data_Sheet_1.docx]

# Supplementary Figures and Tables

**Table 1** Prevalence and frequency of suicide or self - injury among migrant workers (n=2,065)

|  | All participants (n=2,065) | Suicide or self - injury | | *p* | AOR (95% CI) | *p* |
| --- | --- | --- | --- | --- | --- | --- |
|  |  | Yes (n=331) | No (n=1,734) |  |  |  |
| Demographic factors |  |  |  |  |  |  |
| Gender |  |  |  | < 0.001 |  | < 0.001 |
| Male | 885 | 180 (20%) | 705 (80%) |  | 1 (ref) |  |
| Female | 1,180 | 151 (13%) | 1,029 (87%) |  | 0.579 (0.442 - 0.758) |  |
| Age |  |  |  | 0.700 |  | 0.703 |
| 25 or below | 46 | 9 (20%) | 37 (80%) |  | 1 (ref) |  |
| 26 - 35 | 757 | 125 (17%) | 632 (83%) |  | 0.743 (0.315 - 1.752) | 0.743 |
| 36 or above | 1261 | 197 (16%) | 1,064 (84%) |  | 0.605 (0.257 - 1.422) | 0.605 |
| Education level |  |  |  | 0.274 |  | 0.497 |
| Junior high school or below | 46 | 35 (18%) | 11 (82%) |  | 1 (ref) |  |
| High school to Secondary | 757 | 583 (14%) | 174 (86%) |  | 0.854 (0.633 - 1.153) | 0.303 |
| College or above | 1,261 | 931 (10%) | 330 (90%) |  | 1.124 (0.562 - 2.247) | 0.742 |
| Distal factors (childhood ) |  |  |  |  |  |  |
| School dropout |  |  |  | < 0.001 |  | 0.267 |
| No | 1,415 | 1,228 (87%) | 187 (13%) |  | 1 (ref) |  |
| Yes | 650 | 506 (78%) | 144 (22%) |  | 1.180 (0.881 - 1.579) |  |
| Peer victimization |  |  |  | < 0.001 |  | 0.480 |
| No | 129 | 44 (80%) | 85 (20%) |  | 1 (ref) |  |
| Yes | 1,936 | 287 (87%) | 1,649 (13%) |  | 0.846 (0.532 - 1.345) |  |
| Physical abuse/ neglect |  |  |  | < 0.001 |  | < 0.001 |
| No | 1,483 | 163 (11%) | 1,320 (89%) |  | 1 (ref) |  |
| Yes | 582 | 168 (29%) | 414 (71%) |  | 2.517 (1.633 - 3.880) |  |
| Emotional abuse / neglect |  |  |  | < 0.001 |  | 0.465 |
| No | 1,711 | 228 (13%) | 1,483 (87%) |  | 1 (ref) |  |
| Yes | 354 | 103 (29%) | 251 (71%) |  | 1.137 (0.805 - 1.605) |  |
| Proximal factors (adulthood) |  |  |  |  |  |  |
| Work burnout |  |  |  | < 0.001 |  | < 0.001 |
| No | 940 | 58 (6%) | 882 (94%) |  | 1 (ref) |  |
| Yes | 1,125 | 273 (24%) | 852 (76%) |  | 2.196 (1.575 - 3.062) |  |
| Parent - child conflict |  |  |  | < 0.001 |  | < 0.001 |
| No | 710 | 1,182 (88%) | 173 (12%) |  | 1 (ref) |  |
| Yes | 1,355 | 482 (68%) | 228 (32%) |  | 2.517 (1.633 - 3.880) |  |
| Adulthood poverty |  |  |  | < 0.001 |  | 0.003 |
| No | 488 | 20 (4%) | 488 (96%) |  | 1 (ref) |  |
| Yes | 1,577 | 311 (20%) | 1,266 (80%) |  | 2.171 (1.311 - 3.597) |  |
| Divorce intention |  |  |  | < 0.001 |  | < 0.001 |
| No | 1,826 | 220 (12%) | 1,606 (88%) |  | 1 (ref) |  |
| Yes | 239 | 111 (46%) | 128 (54%) |  | 4.540 (3.286 - 6.273) |  |
| Low core self - evaluation |  |  |  | < 0.001 |  | < 0.001 |
| No | 466 | 6 (1%) | 460 (99%) |  | 1 (ref) |  |
| Yes | 1,599 | 325 (20%) | 1,274 (80%) |  | 5.612 (2.401 - 13.116) |  |

**Table 2** Prevalence and frequency of violence among migrant workers (n=2,065)

|  | All participants  (n=2,065) | Violence | | *p* | AOR (95% CI) | *p* |
| --- | --- | --- | --- | --- | --- | --- |
|  |  | Yes (n=1550) | No (n=515) |  |  |  |
| Demographic factors |  |  |  |  |  |  |
| Gender |  |  |  | < 0.001 |  | 0.001 |
| Male | 885 | 706 (80%) | 179 (20%) |  | 1 (ref) |  |
| Female | 1,180 | 844 (72%) | 336 (28%) |  | 0.686 (0.548 - 0.858) |  |
| Age |  |  |  | 0.274 |  | 0.437 |
| 25 or below | 46 | 35 (76%) | 11 (24%) |  | 1 (ref) |  |
| 26 - 35 | 757 | 583 (77%) | 174 (23%) |  | 1.190 (0.560 - 2.531) | 0.651 |
| 36 or above | 1,261 | 931 (73%) | 330 (26%) |  | 1.025 (0.486 - 2.163) | 0.947 |
| Education level |  |  |  | 0.274 |  | < 0.001 |
| Junior high school or below | 1,168 | 852 (73%) | 316 (27%) |  | 1 (ref) |  |
| High school to Secondary | 775 | 600 (77%) | 175 (13%) |  | 1.631 (1.284 - 2.073) | < 0.001 |
| College or above | 122 | 98 (80%) | 24 (20%) |  | 2.653 (1.600 - 4.398) | < 0.001 |
| Distal factors (childhood ) |  |  |  |  |  |  |
| School dropout |  |  |  | < 0.001 |  | 0.003 |
| No | 1,415 | 1,020 (72%) | 395 (28%) |  | 1 (ref) |  |
| Yes | 650 | 530 (81%) | 120 (18%) |  | 1.472 (1.136 - 1.907) |  |
| Peer Bullying/Victimization |  |  |  | 0.033 |  | 0.047 |
| No | 129 | 107 (83%) | 22 (17%) |  | 1 (ref) |  |
| Yes | 1,936 | 1,443 (76%) | 493 (25%) |  | 1.750 (1.008 - 3.040) |  |
| Physical abuse / neglect |  |  |  | < 0.001 |  | 0.002 |
| No | 1,483 | 1,042 (70%) | 441 (30%) |  | 1 (ref) |  |
| Yes | 582 | 508 (87%) | 74 (13%) |  | 1.698 (1.216 - 2.370) |  |
| Emotional abuse / neglect |  |  |  | < 0.001 |  | 0.009 |
| No | 1,711 | 1,235 (72%) | 476 (28%) |  | 1 (ref) |  |
| Yes | 354 | 315 (89%) | 39 (11%) |  | 1.736 (1.150 - 2.620) |  |
| Proximal factors (adulthood) |  |  |  |  |  |  |
| Work burnout |  |  |  | < 0.001 |  | 0.005 |
| No | 940 | 613 (65%) | 327 (35%) |  | 1 (ref) |  |
| Yes | 1,125 | 937 (83%) | 188 (17%) |  | 1.410 (1.107 - 1.795) |  |
| Parent - child conflict |  |  |  | < 0.001 |  | < 0.001 |
| No | 710 | 430 (60%) | 280 (39%) |  | 1 (ref) |  |
| Yes | 1,355 | 1,120 (83%) | 235 (17%) |  | 1.781 (1.397 - 2.270) |  |
| Adulthood poverty |  |  |  | < 0.001 |  | 0.010 |
| No | 488 | 300 (61%) | 188 (39%) |  | 1 (ref) |  |
| Yes | 1,577 | 1,250 (79%) | 327 (21%) |  | 1.386 (1.081 - 1.777) |  |
| Divorce intention |  |  |  | < 0.001 |  | 0.002 |
| No | 1,826 | 1,336 (73%) | 490 (27%) |  | 1 (ref) |  |
| Yes | 239 | 214 (90%) | 25 (10%) |  | 2.010 (1.285 - 3.144) |  |
| Low core self - evaluation |  |  |  | < 0.001 |  | < 0.001 |
| No | 466 | 259 (56%) | 207 (44%) |  | 1 (ref) |  |
| Yes | 1,599 | 1,291 (81%) | 308 (19%) |  | 1.889 (1.448 - 2.465) |  |

**Table 3** Prevalence and frequency of tobacco and alcohol use among migrant workers (n=2,065)

|  | All participants (n=2,065) | tobacco and alcohol use | | *p* | AOR (95% CI) | *p* |
| --- | --- | --- | --- | --- | --- | --- |
|  |  | Yes (n=1,550) | No (n=515) |  |  |  |
| Demographic factors |  |  |  |  |  |  |
| Gender |  |  |  | < 0.001 |  | 0.001 |
| Male | 885 | 687 (78%) | 198 (22%) |  | 1 (ref) |  |
| Female | 1,180 | 349 (30%) | 831 (70%) |  | 0.097 (0.077 - 0.122) |  |
| Age |  |  |  | 0.13 |  | 0.084 |
| 25 or below | 46 | 19 (41%) | 27 (58%) |  | 1 (ref) |  |
| 26 - 35 | 757 | 399 (53%) | 358 (47%) |  | 1.424 (0.688 - 2.951) | 0.341 |
| 36 or above | 1,261 | 618 (49%) | 643 (51%) |  | 1.115 (0.541 - 2.298) | 0.769 |
| Education level |  |  |  | 0.437 |  | 0.536 |
| Junior high school or below | 1,168 | 580 (50%) | 588 (50%) |  | 1 (ref) |  |
| High school to Secondary | 775 | 400 (52%) | 375 (48%) |  | 1.140 (0.903 - 1.440) | 0.271 |
| College or above | 122 | 56 (46%) | 66 (54%) |  | 1.117 (0.702 - 1.777) | 0.641 |
| Distal factors (childhood ) |  |  |  |  |  |  |
| School dropout |  |  |  | < 0.001 |  | 0.053 |
| No | 1,415 | 655 (46%) | 760 (54%) |  | 1 (ref) |  |
| Yes | 650 | 381 (59%) | 269 (41%) |  | 1.271 (0.997 - 1.621) |  |
| Peer victimization |  |  |  | < 0.001 |  | 0.162 |
| No | 129 | 89 (69%) | 40 (31%) |  | 1 (ref) |  |
| Yes | 1,936 | 947 (49%) | 989 (51%) |  | 0.711 (0.441 - 1.146) |  |
| Physical abuse / neglect |  |  |  | < 0.001 |  | 0.140 |
| No | 1,483 | 656 (44%) | 827 (58%) |  | 1 (ref) |  |
| Yes | 582 | 380 (65%) | 202 (35%) |  | 1.237 (0.932 - 1.643) |  |
| Emotional abuse / neglect |  |  |  | < 0.001 |  | 0.018 |
| No | 1,711 | 797 (47%) | 914 (53%) |  | 1 (ref) |  |
| Yes | 354 | 239 (68%) | 115 (32%) |  | 1.484 (1.071 - 2.056) |  |
| Proximal factors (adulthood) |  |  |  |  |  |  |
| Work burnout |  |  |  | < 0.001 |  | < 0.001 |
| No | 940 | 353 (38%) | 587 (62%) |  | 1 (ref) |  |
| Yes | 1,125 | 683 (61%) | 442 (39%) |  | 1.694 (1.341 - 2.141) |  |
| Parent - child conflict |  |  |  | < 0.001 |  | < 0.001 |
| No | 710 | 252 (35%) | 458 (65%) |  | 1 (ref) |  |
| Yes | 1,355 | 784 (58%) | 571 (42%) |  | 1.726 (1.341 - 2.223) |  |
| Adulthood poverty |  |  |  | < 0.001 |  | < 0.001 |
| No | 488 | 156 (32%) | 332 (68%) |  | 1 (ref) |  |
| Yes | 1,577 | 880 (59%) | 697 (44%) |  | 1.792 (1.366 - 2.351) |  |
| Divorce intention |  |  |  | < 0.001 |  | < 0.001 |
| No | 1,826 | 873 (48%) | 953 (52%) |  | 1 (ref) |  |
| Yes | 239 | 163 (68%) | 76 (31%) |  | 2.221 (1.581 - 3.121) |  |
| Low core self - evaluation |  |  |  | < 0.001 |  | 0.031 |
| No | 466 | 159 (34%) | 307 (66%) |  | 1 (ref) |  |
| Yes | 1,599 | 877 (55%) | 722 (45%) |  | 1.387 (1.03 - 1.867) |  |

**Table 4** Prevalence and frequency of breaking discipline among migrant workers (n=2,065)

|  | All participants (n=2,065) | breaking discipline | | *p* | AOR (95% CI) | *p* |
| --- | --- | --- | --- | --- | --- | --- |
|  |  | Yes (n=447) | No (n=1,618) |  |  |  |
| Demographic factors |  |  |  |  |  |  |
| Gender |  |  |  | < 0.001 |  | < 0.001 |
| Male | 885 | 290 (33%) | 595 (67%) |  | 1 (ref) |  |
| Female | 1,180 | 157 (13%) | 1023 (87%) |  | 0.297 (0.233 - 0.378) |  |
| Age |  |  |  | 0.298 |  | 0.461 |
| 25 or below | 46 | 6 (13%) | 40 (87%) |  | 1 (ref) |  |
| 26 - 35 | 757 | 160 (21%) | 597 (79%) |  | 1.819 (0.689 - 4.806) | 0.227 |
| 36 or above | 1,261 | 281 (22%) | 980 (78%) |  | 1.848 (0.703 - 4.861) | 0.213 |
| Education level |  |  |  | 0.107 |  | 0.860 |
| Junior high school or below | 1,168 | 266 (23%) | 902 (77%) |  | 1 (ref) |  |
| High school to Secondary | 775 | 163 (21%) | 612 (79%) |  | 1.024 (0.787 - 1.333) | 0.858 |
| College or above | 122 | 18 (15%) | 104 (85%) |  | 1.176 (0.658 - 2.103) | 0.584 |
| Distal factors (childhood) |  |  |  |  |  |  |
| School dropout |  |  |  | < 0.001 |  | 0.115 |
| No | 1,415 | 258 (18%) | 1157 (82%) |  | 1 (ref) |  |
| Yes | 650 | 189 (29%) | 461 (71%) |  | 1.235 (0.95 - 1.604) |  |
| Peer victimization |  |  |  | < 0.001 |  | 0.606 |
| No | 129 | 52 (40%) | 77 (60%) |  | 1 (ref) |  |
| Yes | 1,936 | 395 (20%) | 1541 (80%) |  | 0.89 (0.572 - 1.386) |  |
| Physical Abuse / Neglect |  |  |  | < 0.001 |  | 0.003 |
| No | 1,483 | 231 (16%) | 1252 (84%) |  | 1 (ref) |  |
| Yes | 582 | 216 (37%) | 366 (63%) |  | 1.546 (1.162 - 2.058) |  |
| Emotional Abuse / Neglect |  |  |  | < 0.001 |  | 0.001 |
| No | 1,711 | 302 (18%) | 1409 (82%) |  | 1 (ref) |  |
| Yes | 354 | 145 (41%) | 209 (59%) |  | 1.712 (1.253 - 2.338) |  |
| Proximal factors (adulthood) |  |  |  |  |  |  |
| Work burnout |  |  |  | < 0.001 |  | 0.002 |
| No | 940 | 111 (12%) | 829 (88%) |  | 1 (ref) |  |
| Yes | 1,125 | 336 (30%) | 789 (70%) |  | 1.541 (1.171 - 2.027) |  |
| Parent - child conflict |  |  |  | < 0.001 |  | < 0.001 |
| No | 710 | 59 (8%) | 651 (91%) |  | 1 (ref) |  |
| Yes | 1,355 | 388 ( (29%) | 967 (71%) |  | 2.161 (1.552 - 3.01) |  |
| Adulthood poverty |  |  |  | < 0.001 |  | 0.004 |
| No | 488 | 42 (9%) | 446 (91%) |  | 1 (ref) |  |
| Yes | 1,577 | 405 (26%) | 1172 (74%) |  | 1.742 (1.194 - 2.54) |  |
| Divorce intention |  |  |  | < 0.001 |  | < 0.001 |
| No | 1,826 | 338 (19%) | 1,488 (81%) |  | 1 (ref) |  |
| Yes | 239 | 109 (46%) | 130 (54%) |  | 2.794 (2.028 - 3.85) |  |
| Low core self - evaluation |  |  |  | < 0.001 |  | 0.003 |
| No | 466 | 32 (7%) | 434 (93%) |  | 1 (ref) |  |
| Yes | 1599 | 415 (26%) | 1,184 (74%) |  | 1.927 (1.252 - 2.966) |  |

**Supplementary Figure**

**
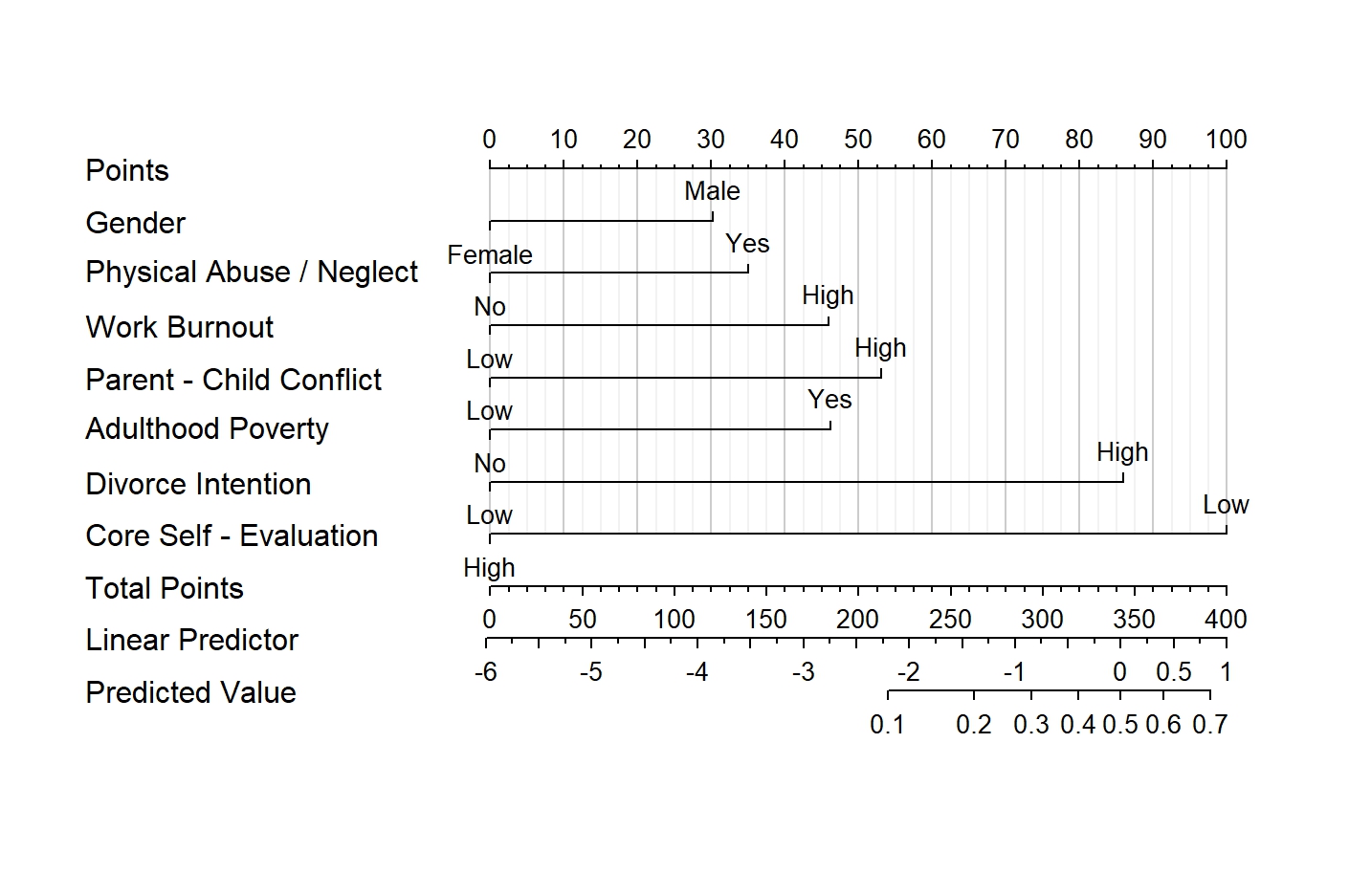
**

**Fig. 1** Proposed nomogram for predicting the probability of suicide or self - injury.

| 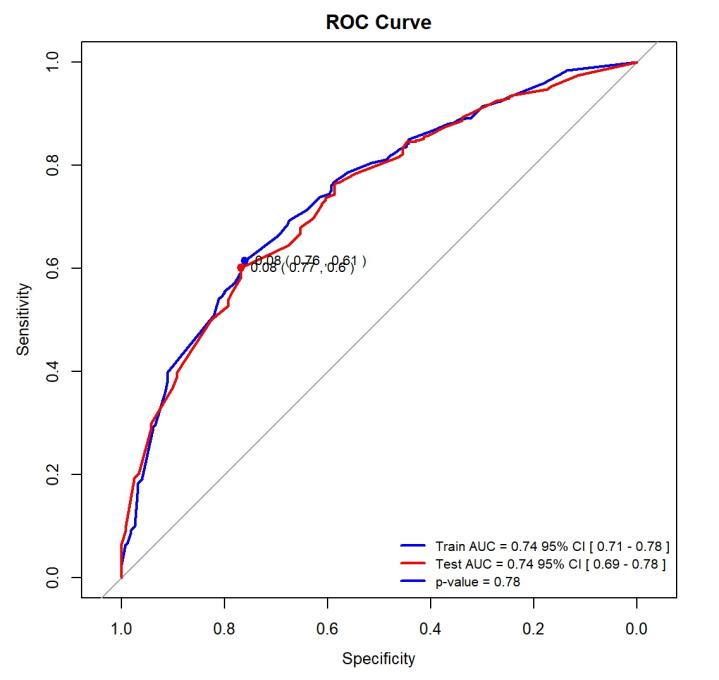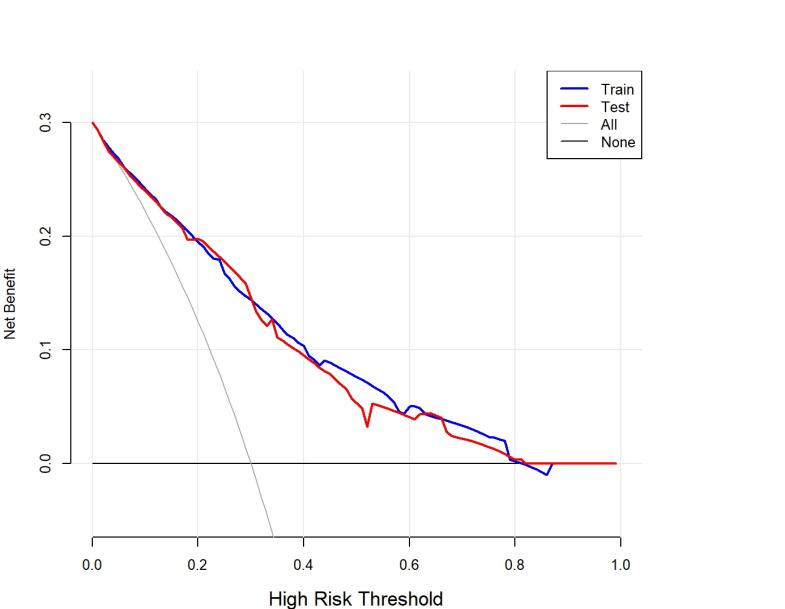  (**A)** (**B)**  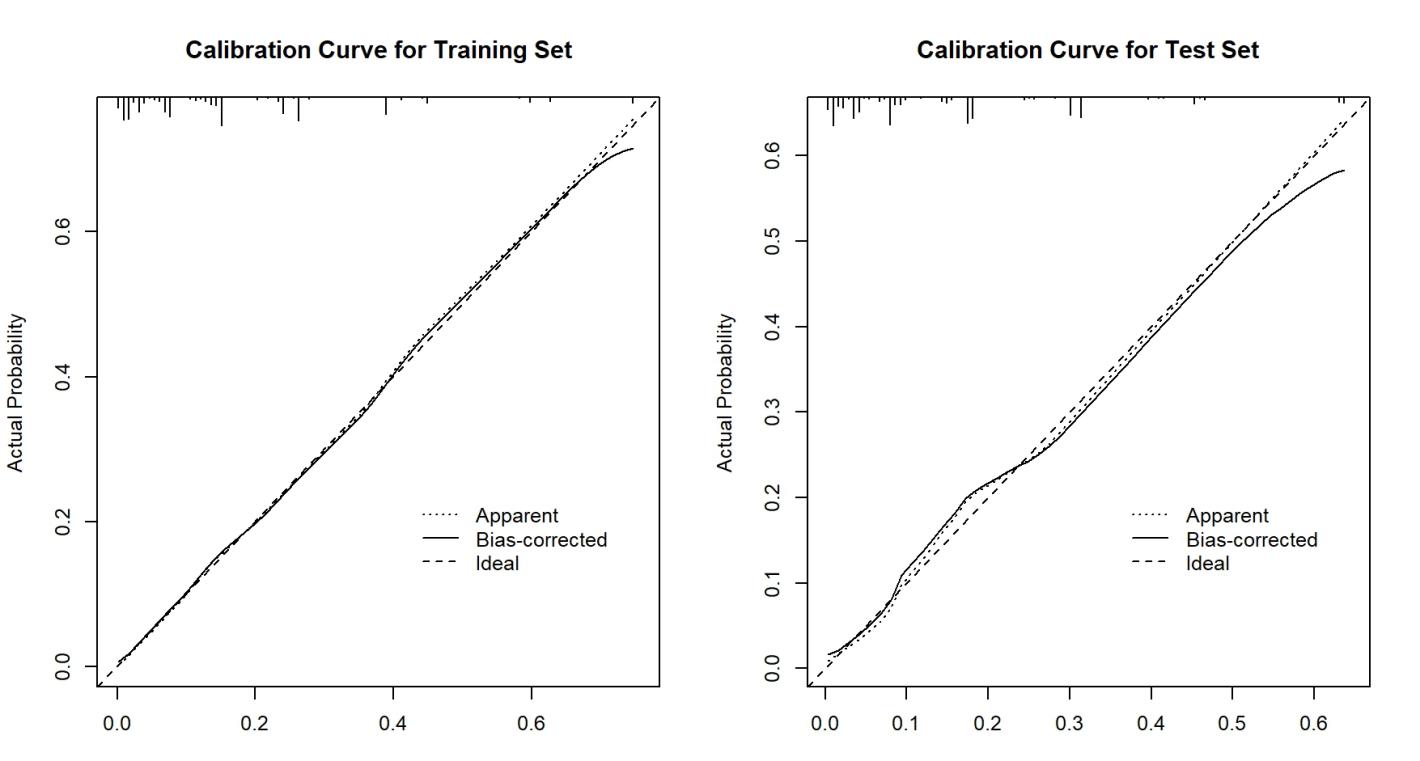  (**C_1_**) (**C_2_**)  **Fig. 2** Validation of the nomograms. **(A)** ROC curves of the study’s generated nomogram for predicting the probability of suiside or self - injury. **(B)** DCA for predicting the probability of suicide or self - injury’s nomogram. **(C)** Calibration curves of the nomogram for predicting the probability of suicide or self - injury: **(C_1_)** for the training set; and **(C_2_)** for the internal validation. |
| --- |


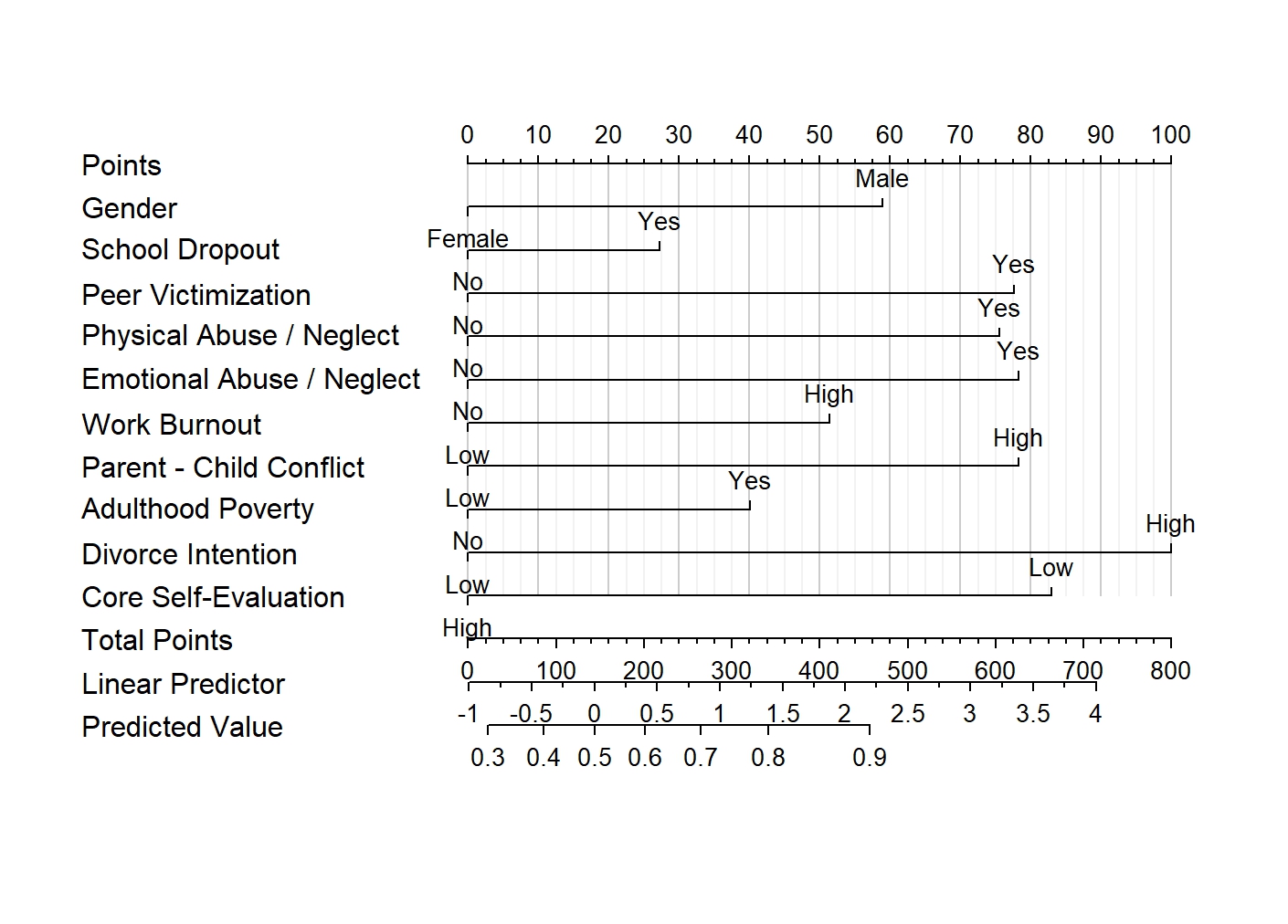


**Fig.3** Proposed nomogram for predicting the probability of violence

| 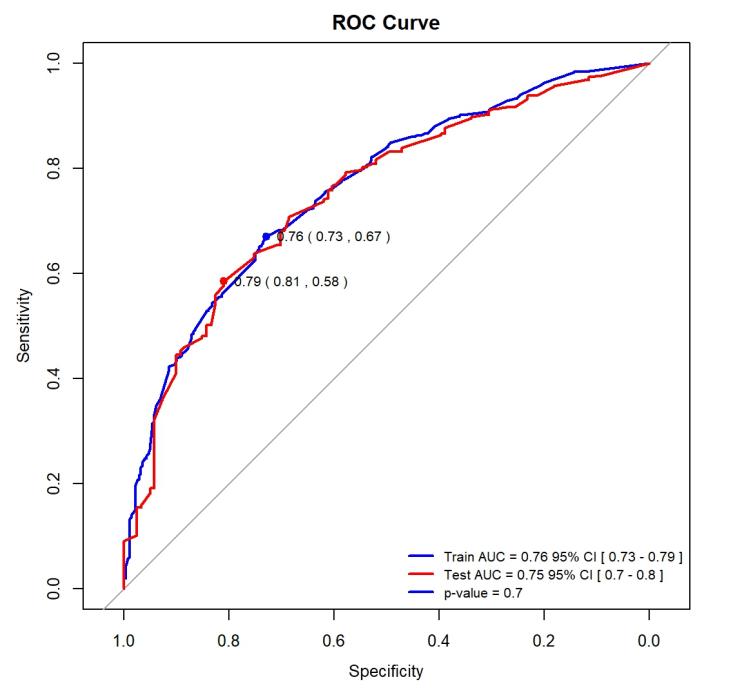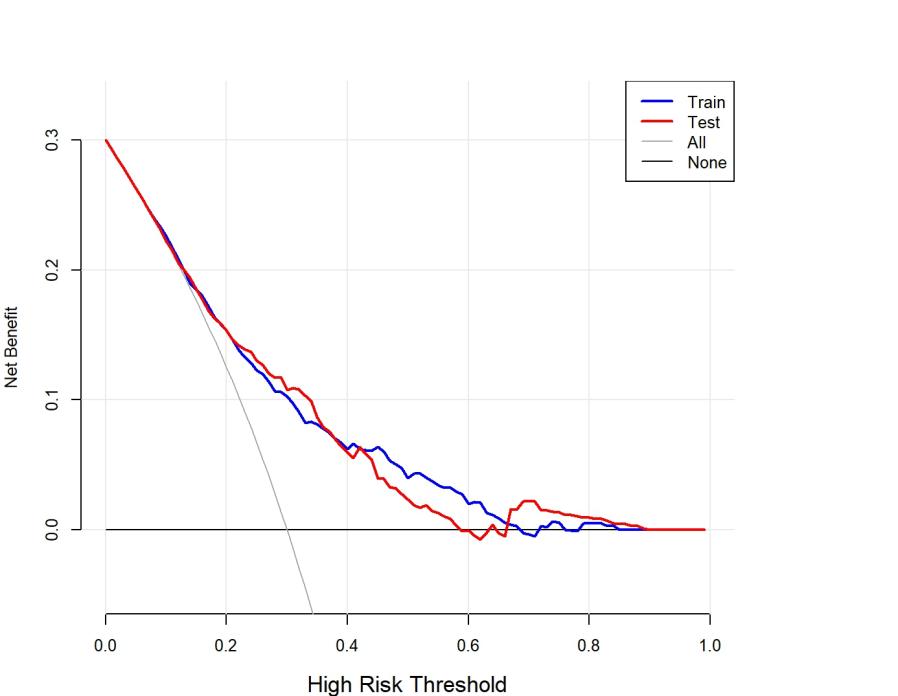  (**A) (B)**  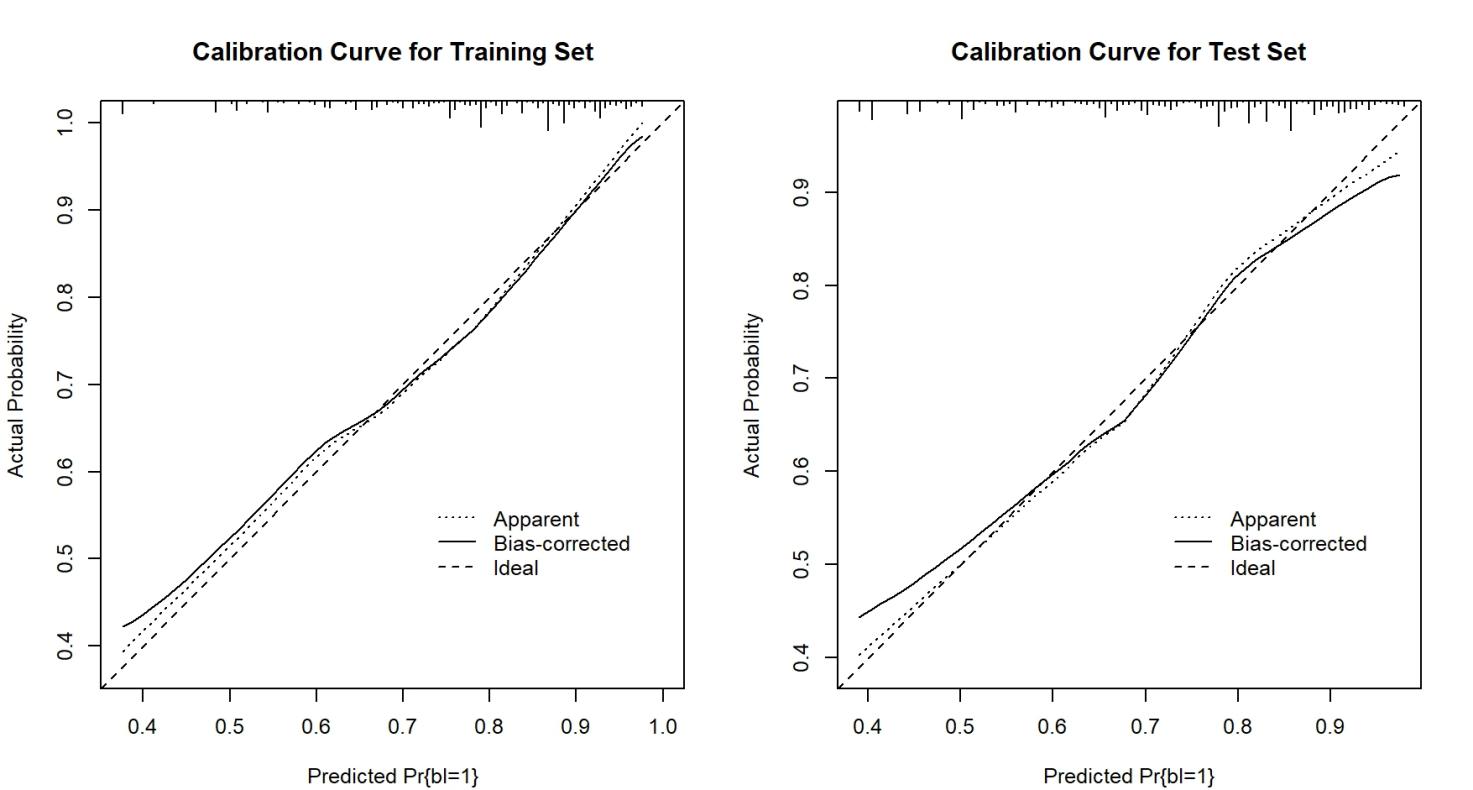  **(C_1_) (C_2_)**  **Fig. 4** Validation of the nomograms. **(A)** ROC curves of the study’s generated nomogram  for predicting the probability of violence. **(B)** DCA for predicting the probability of violence’s nomogram. **(C)** Calibration curves of the nomogram for predicting the probability of violence: **(C_1_)** for the training set; and **(C_2_)** for the internal validation. |
| --- |


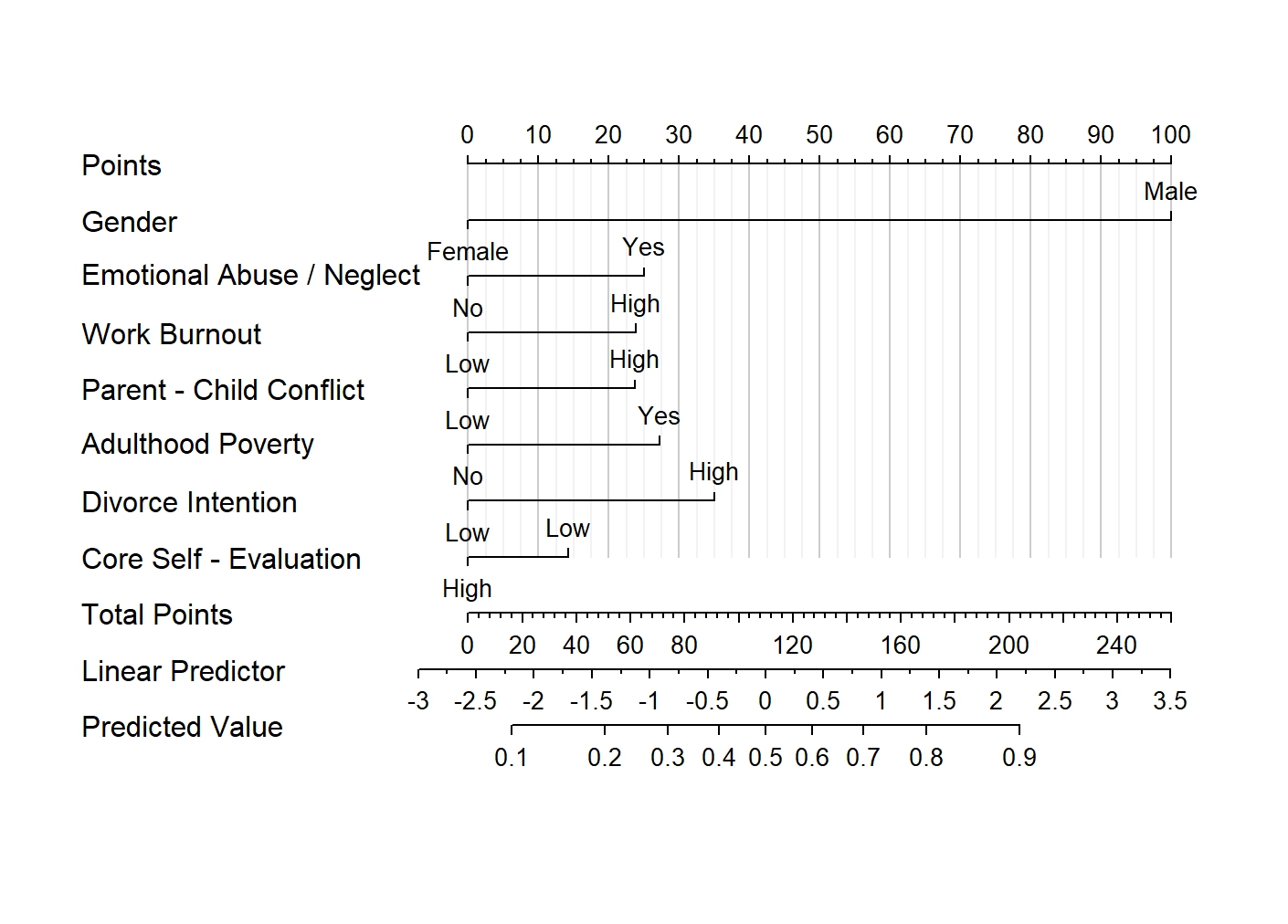


**Fig. 5** Proposed nomogram for predicting the probability of tobacco and alcohol use.

| 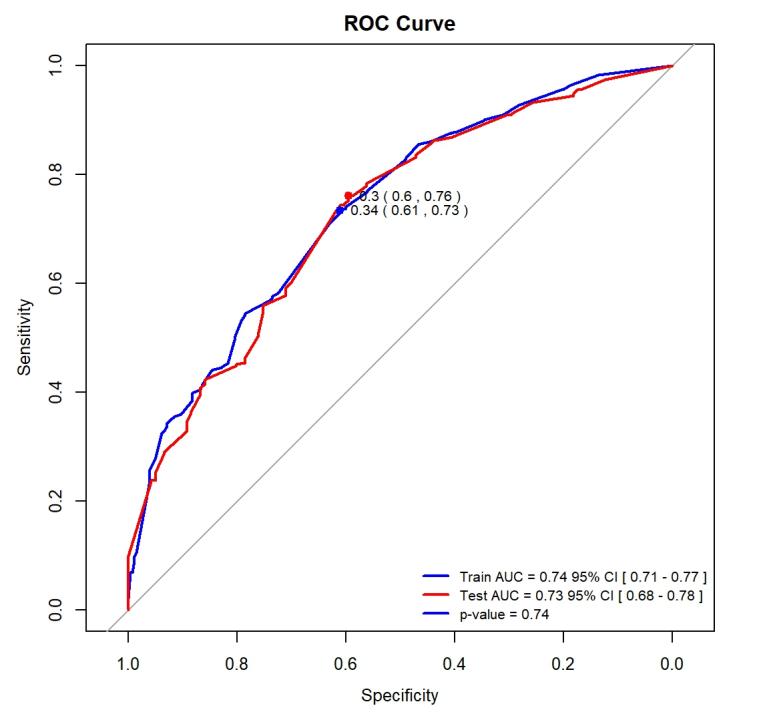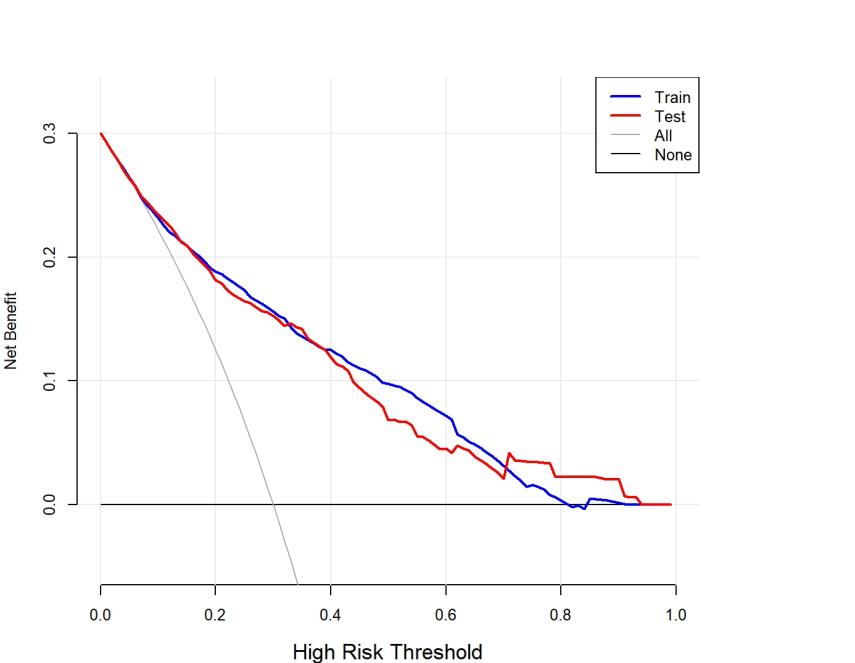  **(A) (B)**  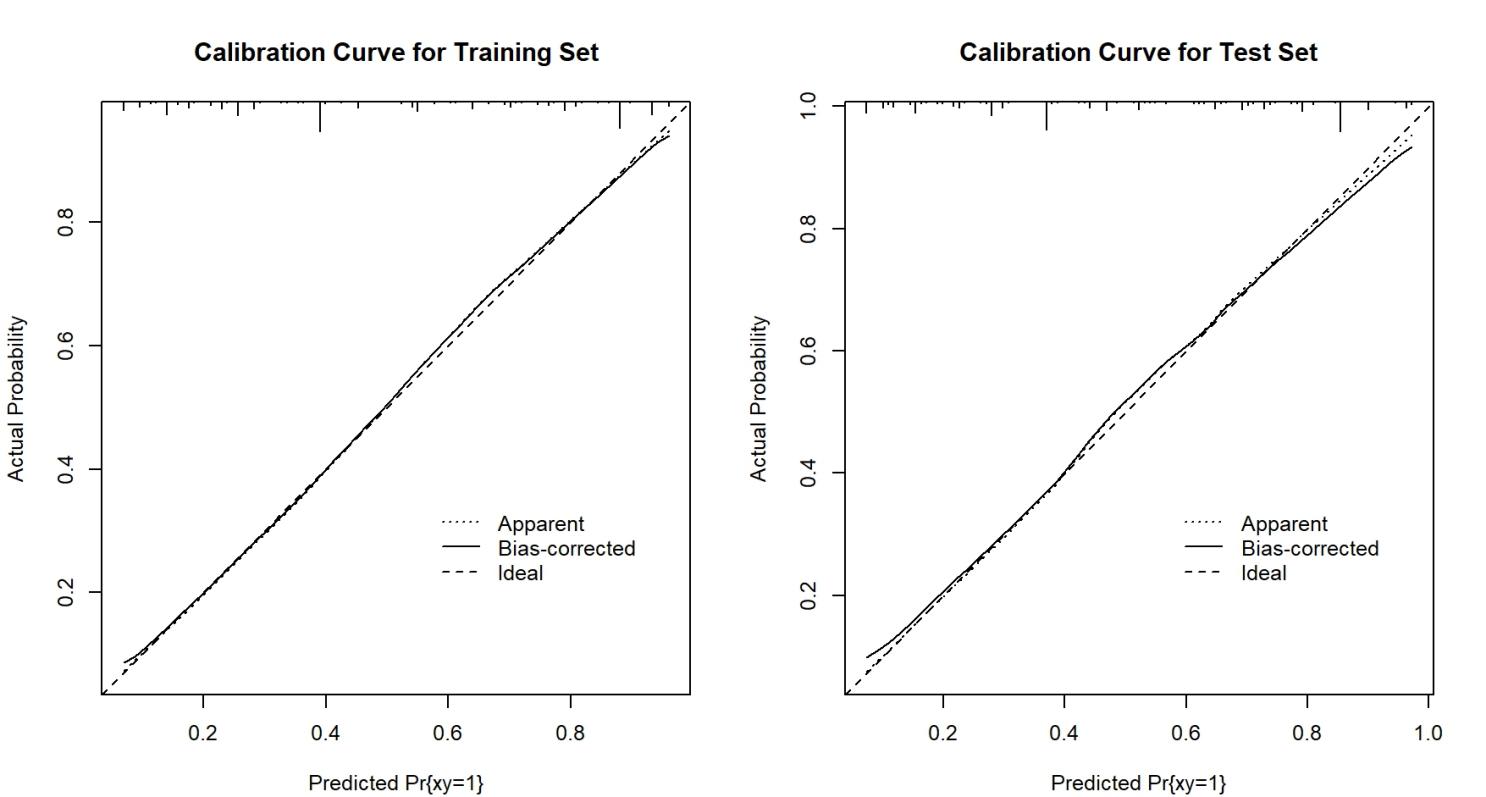  **(C_1_) (C_2_)**  **Fig. 6** Validation of the nomograms. **(A)** ROC curves of the study’s generated nomogram for predicting the probability of tobacco and alcohol use. **(B)** DCA for predicting the probability of tobacco and alcohol use’s nomogram. **(C)** Calibration curves of the nomogram for predicting the probability of tobacco and alcohol use : **(C_1_)** for the training set; and **(C_2_)** for the internal validation. |
| --- |


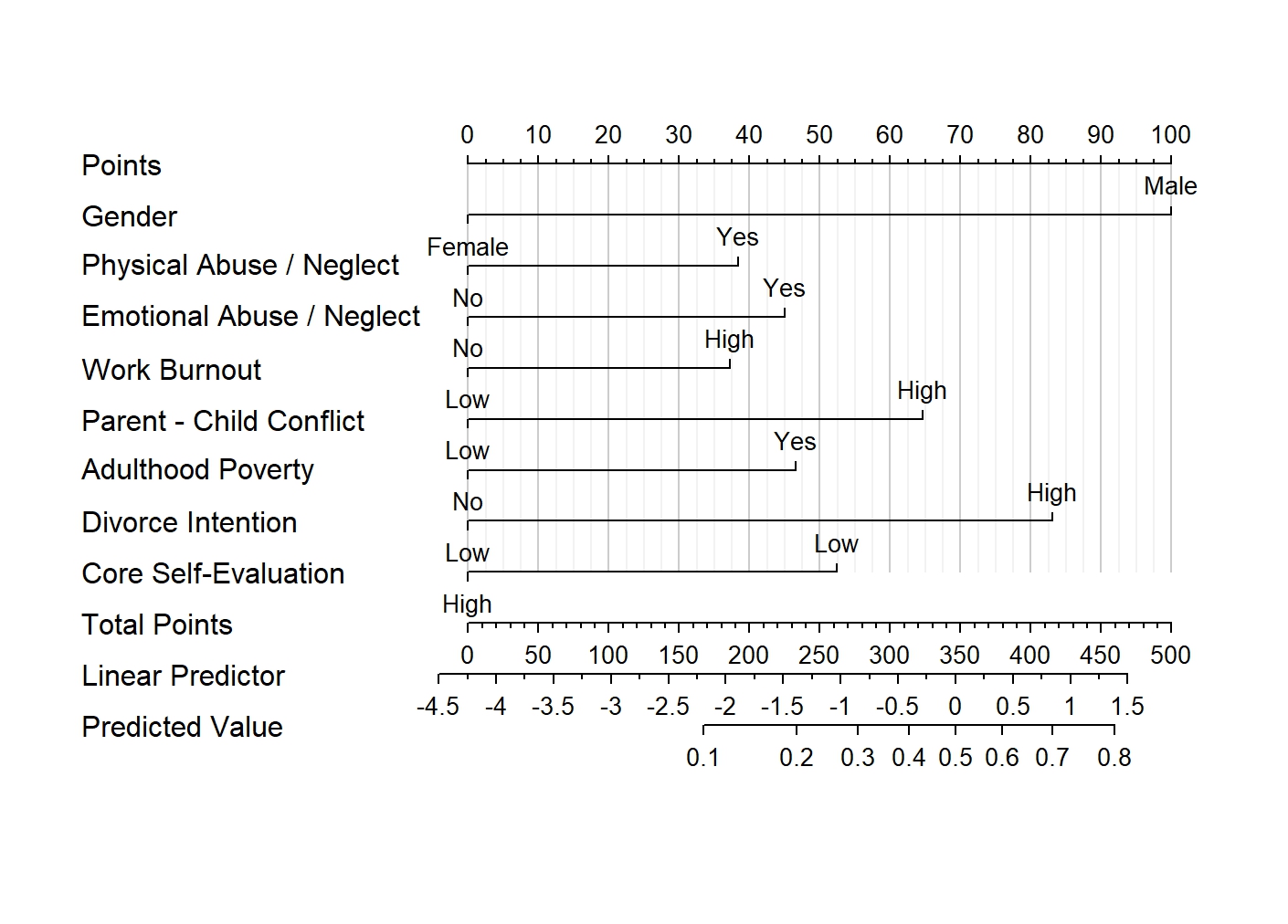


**Fig. 7** Proposed nomogram for predicting the probability of breaking discipline.

| **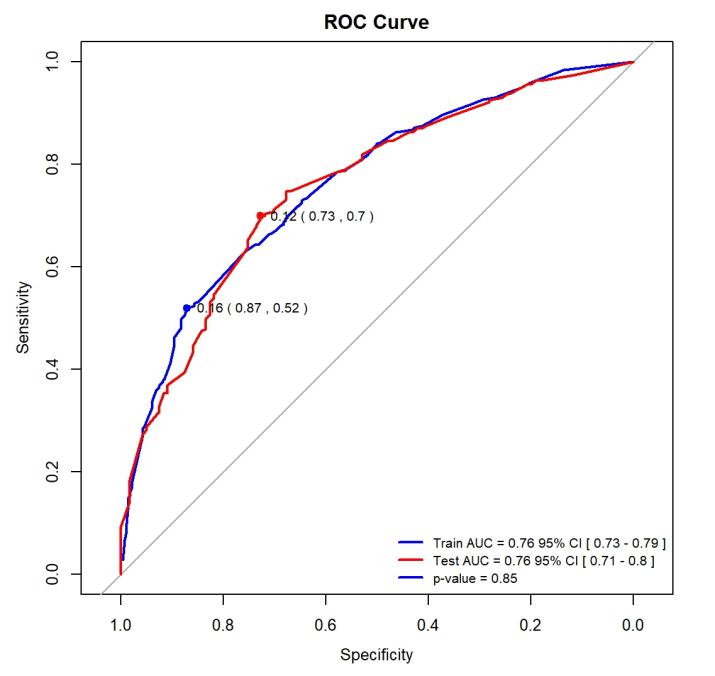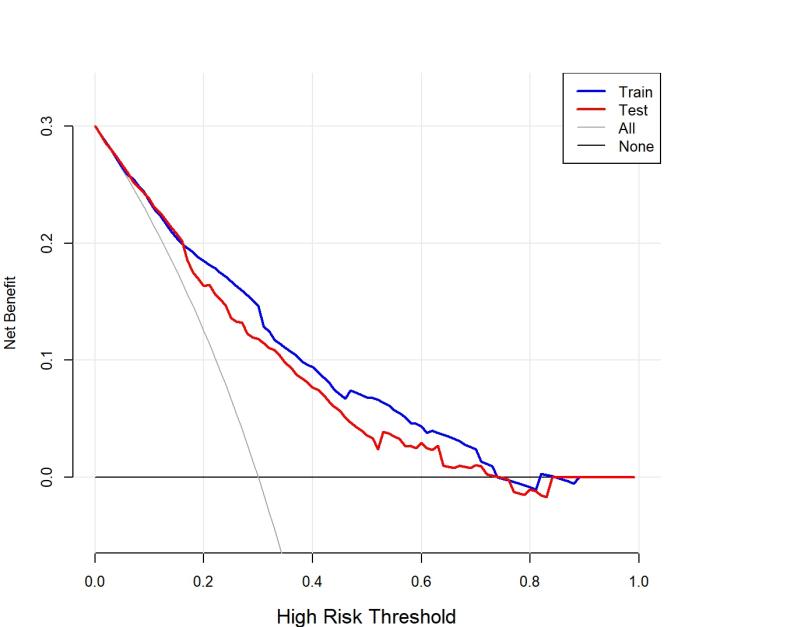 (A) (B)**  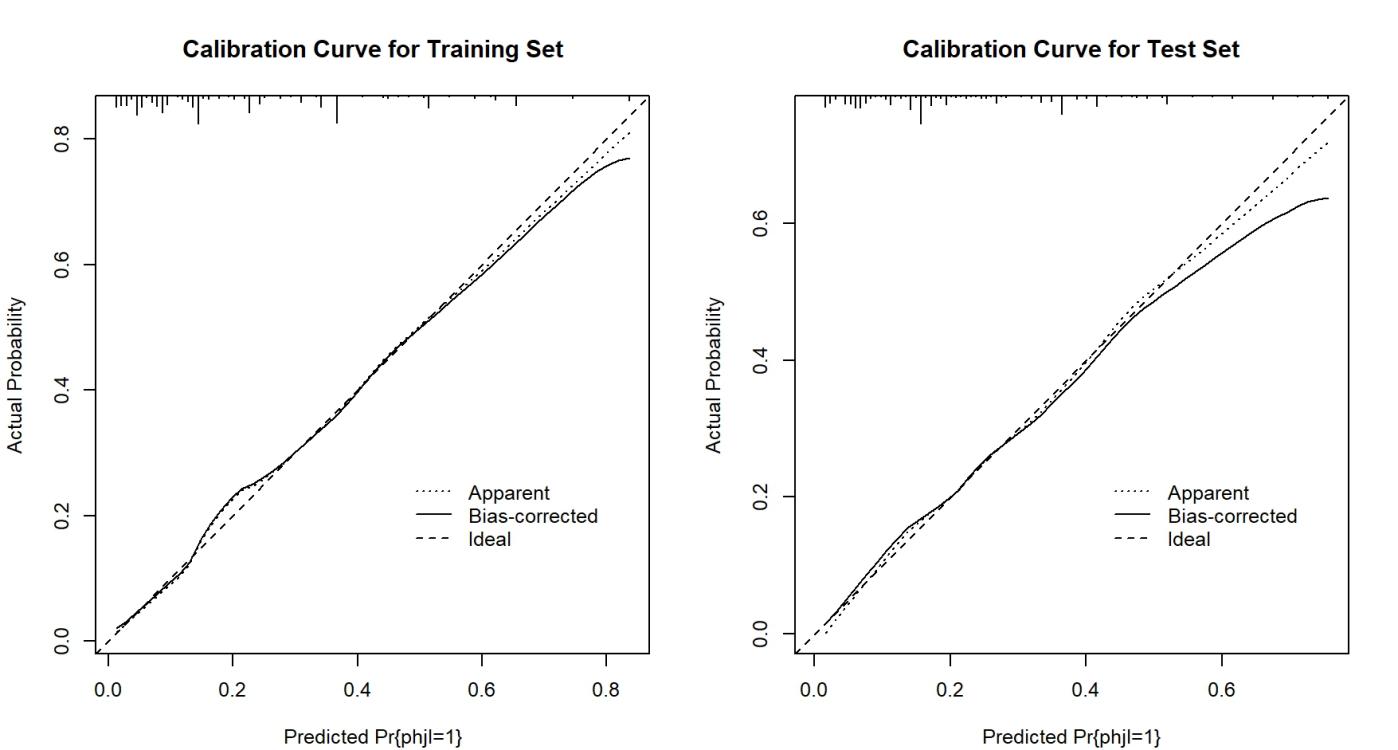  **(C_1_) (C_2_)**  **Fig. 8** Validation of the nomograms. **(A)** ROC curves of the study’s generated nomogram for predicting the probability of breaking discipline. **(B)** DCA for predicting the probability of breaking discipline’s nomogram. **(C)** Calibration curves of the nomogram for predicting the probability of breaking discipline :**(C_1_)** for the training set; and **(C_2_)** for the internal validation. |
| --- |
